# Comparative proteomic analysis of cat eye syndrome critical region protein 1- function in tumor-associated macrophages and immune response regulation of glial tumors

**DOI:** 10.18632/oncotarget.26063

**Published:** 2018-09-11

**Authors:** Changbin Zhu, Dana A.M. Mustafa, Merle M. Krebber, Ihsan Chrifi, Pieter J.M. Leenen, Dirk J. Duncker, Lennard Dekker, Theo M. Luider, Johan M. Kros, Caroline Cheng

**Affiliations:** ^1^ Department of Pathology, Erasmus Medical Center, Rotterdam, The Netherlands; ^2^ Division of Experimental Cardiology, Department of Cardiology, Erasmus Medical Center, Rotterdam, The Netherlands; ^3^ Department of Immunology, Erasmus Medical Center, Rotterdam, The Netherlands; ^4^ Department of Neurology, Erasmus Medical Center, Rotterdam, The Netherlands; ^5^ Department of Nephrology and Hypertension, DIGD, University Medical Center Utrecht, Utrecht, The Netherlands; ^6^ Department of Paediatric Neurosurgery, Shanghai Xin Hua Hospital/Shanghai Jiao Tong University School of Medicine, Shanghai, PR China

**Keywords:** tumor associated macrophages, glioma, CECR1, proteomics, immune response

## Abstract

**Introduction:**

Tumor associated macrophages (TAMs) promote tumor development, angiogenesis and distal metastasis. In previous studies, we showed that Cat Eye Syndrome Critical Region Protein 1 (CECR1) is expressed by M2-like TAMs in human glioma samples. CECR1 promoted M2 TAMs differentiation and affected glioma cell proliferation and migration. Here we investigated the proteomic profile of TAMs expressing CECR1 in absence or presence of glioma cells.

**Results:**

CECR1 siRNA transfection upregulated 67 proteins in THP-1-derived Macrophages (MQs). Pathway annotation mapped this set to 3 major pathways relevant for MQ function, including ‘MHC-I antigen presentation’, ‘phagosome maturation’ and ‘endocytosis’. Co-culture of siCECR1 THP-1-derived MQs with U87 glioma cells attenuated the changes observed on protein and mRNA level in response to MQ CECR1 silencing. SiCECR1 in U87 co-cultured MQs was associated with an IL-10^low^, IL-12^high^ M1-like phenotype. In U87 co-culture conditions, SiCECR1 also downregulated S20 proteasome complex proteins PSMA5, PSMA7, PSMC6 and PSMD8. This protein profile was linked to a low proliferation rate of siCECR1 MQs. Overlap analysis identified S100A9 and PLAU as CECR1-related proteins that were significantly correlated with expression of CECR1 and macrophage lineage markers in three large public GBM datasets.

**Conclusion:**

This study reports the molecular pathways and key molecules that are mediated by CECR1 function in THP- 1-derived MQs and TAMs in glioma.

**Methods:**

PMA-treated THP-1 cells (MQs) were siRNA transfected for CECR1 *in vitro*, with or without stimulation of the primary glioma cell line U87. Lysates were analyzed by (nano)LC-MS. Significant altered protein levels were identified (*P* < 0.05), followed by pathway annotation.

## INTRODUCTION

Glial tumors are located in the immune privileged environment of the central nervous system. They are highly heterogeneous in cell composition, limiting effective therapies. In glioblastoma multiforme (GBM), the proportions of mesenchymal cells including macrophages and microglia can comprise up to 40% of all cells [1]. Macrophages (MQs) are a major component of the innate immune system [2] and their presence in glial tumors suggests they have a role in tumor pathogenesis and may influence the success of treatment [3, 4]. Due to their relatively high plasticity, MQs can adapt to local microenvironments by differentiating into sub-phenotypes with various functions [5, 6]. The immune-phenotypes of MQs are, for practical reasons, commonly classified into either the M1 and M2 spectrum [7]. However, it is acknowledged that the strict dichotomy does not reflect the actual immune profile of the cells; there is a gradual transition between M1 and M2 and other subtypes may exist as well. In the microenvironment of malignant tumors, recruited monocytes predominantly differentiate into tumor-associated macrophages (TAMs), hereby typically exhibiting an M2-like MQ phenotype. M2-like TAMs are characterized by higher expression levels of cell surface markers CD163, CD204, CD206, and CSFR1. In addition, M2-like TAMs secrete higher levels of IL-10, CCL18, TGF-β, and COX-2 [8]. This paracrine profile mainly induces local immune-suppression by inhibiting infiltration of CD8+ T cells and promoting activation of the regulatory T cells population [9]. TAMs also promote tumor angiogenesis via secretion of pro-angiogenic factors and enzymes like VEGFA, VEGFC, PDGFB, PDGFC, uPA, FGF, Cathepsin and MMPs [10, 11]. In breast, prostate, bladder and cervical cancers, the presence of M2-like TAMs is therefore strongly associated with an immunosuppressive tumor microenvironment, increased malignancy and poor prognosis [12–14]. The number of TAMs is moreover highly correlated with human glioma vascular density [15].

A better understanding of the molecular regulators of M2-like TAMs will improve the identification of new drug targets in the treatment of GBM and other types of malignant tumors. In previous studies of diffuse gliomas, we examined the function of cat eye syndrome critical region 1 (CECR1), a conserved molecule that is highly expressed in the macrophage lineage [16]. CECR1 reportedly functions as a growth factor and immune regulator through its adenosine deaminase enzymatic activity as well as by direct binding to adenosine receptors in invertebrates and vertebrates [17–19]. More specifically, CECR1 binds to adenosine receptors and functions as a growth factor for monocytes and T lymphocytes via autocrine and paracrine stimulation [20]. In monocytes, CECR1 has been shown to promote MQ differentiation [20]. In a recent genetic study, loss-of-function mutations in CECR1 were associated with a range of vascular and inflammatory phenotypes in patients with syndromic presentations of early-onset stroke, systemic vasculopathy and auto-inflammatory diseases [21]. The cytokine profile of patient-derived CECR1 gene deficient MQs demonstrated a predominant M1-like pro-inflammatory phenotype [21, 22]. Our previous findings reported an increased expression of CECR1 in TAMs of gliomas, and provided causal evidence that CECR1 is vital for promoting TAM differentiation towards a M2-like (immune-suppressive) phenotype [23]. Furthermore, we demonstrated that CECR1 functions as an oncogenic molecule that enhances glioma proliferation, migration and angiogenesis via direct crosstalk between TAMs and glial cells [23].

Although these studies improved our understanding of CECR1 function in TAMs, the complex intracellular molecular mechanisms in MQs that are mediated by CECR1, remain to be further elucidated. In this study, we aimed to define the function of CECR1 in TAMs by a proteomic approach. We identified key CECR1 regulated molecules and pathways in normal macrophages and TAMs that are activated by glial tumor cells.

## RESULTS

### Changes in proteomic profile of THP-1 MQs in response to CECR1 siRNA silencing

**Figure 1:**
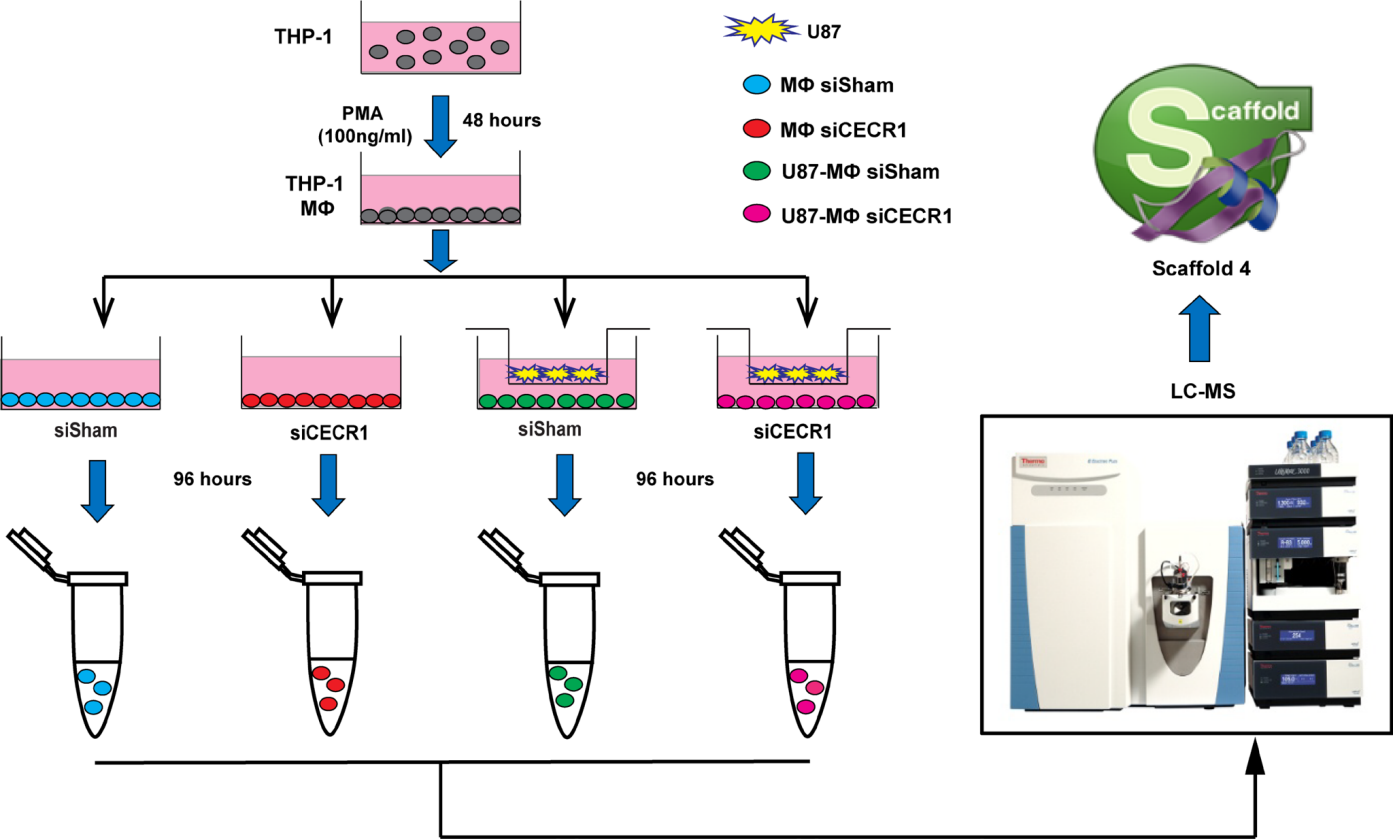
Schematic illustration of study design and workflow Human monocytic THP-1 cells were differentiated into macrophages (MQs) by PMA stimulation (100 ng/ml) for 48 hours followed by siRNA transfection (CECR1 targeting versus non-targeted scrambled siSham as transfection control, using two different siRNA and scrambled systems). Glioblastoma cell line U87 cells were co-cultured with THP-1 derived MQs with/without siCECR1 in a separate chamber with a 0.4-micron pore size filter to enable paracrine interaction between the two cell types. After 96 hours of incubation, cell lysates of were harvested and analyzed by nano-LC/MS. Raw data was imported into Scaffold 4 for processing and exported to Excel for further analysis.

A flow chart of the study design is provided in Figure 1. For proteomic analysis, CECR1 was silenced in PMA-treated THP-1 cells (MQs) by siRNA for 96 hours. The efficiency of CECR1 silencing was validated using two separate siRNA approaches; 1) by immunofluorescent visualization of the CECR1 signal in siCECR1 treated MQs compared with non-targeting scrambled siRNA (siSham, Dharmacon) transfected control MQs and 2) by immunofluorescent visualization of the CECR1 signal with a second set of non-overlapping siCECR treated MQs compared to a fluorescently labeled universal negative control (siSham, Sigma Aldrich) (Supplementary Figure 1A–1D).

**Figure 2:**
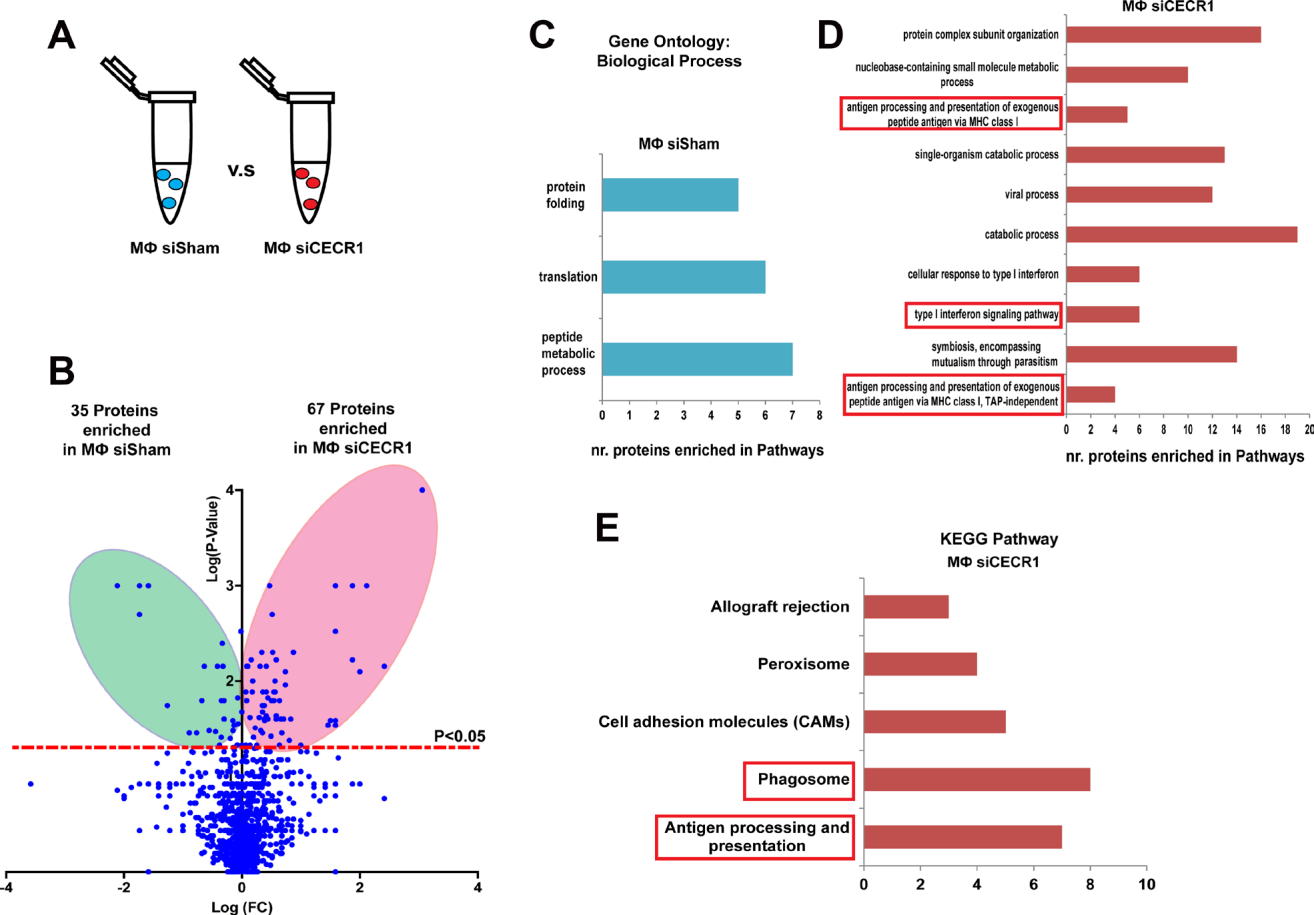
Changes in proteomic profile of THP-1 macrophages in response to CECR1 silencing (**A**) Schematic illustration displaying the study design of comparing siCECR1 and siSham transfected THP-1 derived MQs. (**B**) Volcano blot showing differentially expressed proteins in siSham and siCECR1 transfected MQs. Proteins were considered differentially expressed if *P*-value < 0.05. Expression levels of proteins are displayed on X-axis in log fold change. *P*-values are displayed on Y-axis log fold. (**C**) Bar graph displaying the annotation results of Gene Ontology. Shown are the Biological processes with FDR (False Discovery Rate) < 0.05 that are enriched in siSham compared to siCECR1 transfected MQs. X-axis displays the number of input proteins in each biological process. (**D**) Bar graph displaying the top 10 annotations of Gene Ontology with FDR < 0.05 that are enriched in siCECR1 compared with siSham transfected MQs. X-axis displays number of input proteins in each annotation. (**E**) Bar graph showing the top 5 KEGG pathways that are enriched in siCECR1 versus siSham transfected MQs. X-axis displays number of input proteins in each pathway.

Protein lysates derived from CECR1-silenced (Dharmacon) versus siSham treated MQs were analyzed by mass spectrometry (Figure 2A). In total, 102 proteins were affected by CECR1 silencing (Supplementary Tables 1 and 2). Thirty-five proteins were downregulated (more enriched in siSham group) and 67 proteins were upregulated in the CECR1-silenced group (more enriched in the siCECR1 group (Figure 2B). For validation, RNA expression of 5 top proteins upregulated in siCECR1 vs. siSham, EVL, HLA-C, ITGB7, SEPT7 and GALM, was investigated using the second non-overlapping siCECR1 system. In line with the proteomics analysis, RNA expression significantly increased in response to siCECR1, except for GALM (Supplementary Figure 1E–1I).

**Figure 3:**
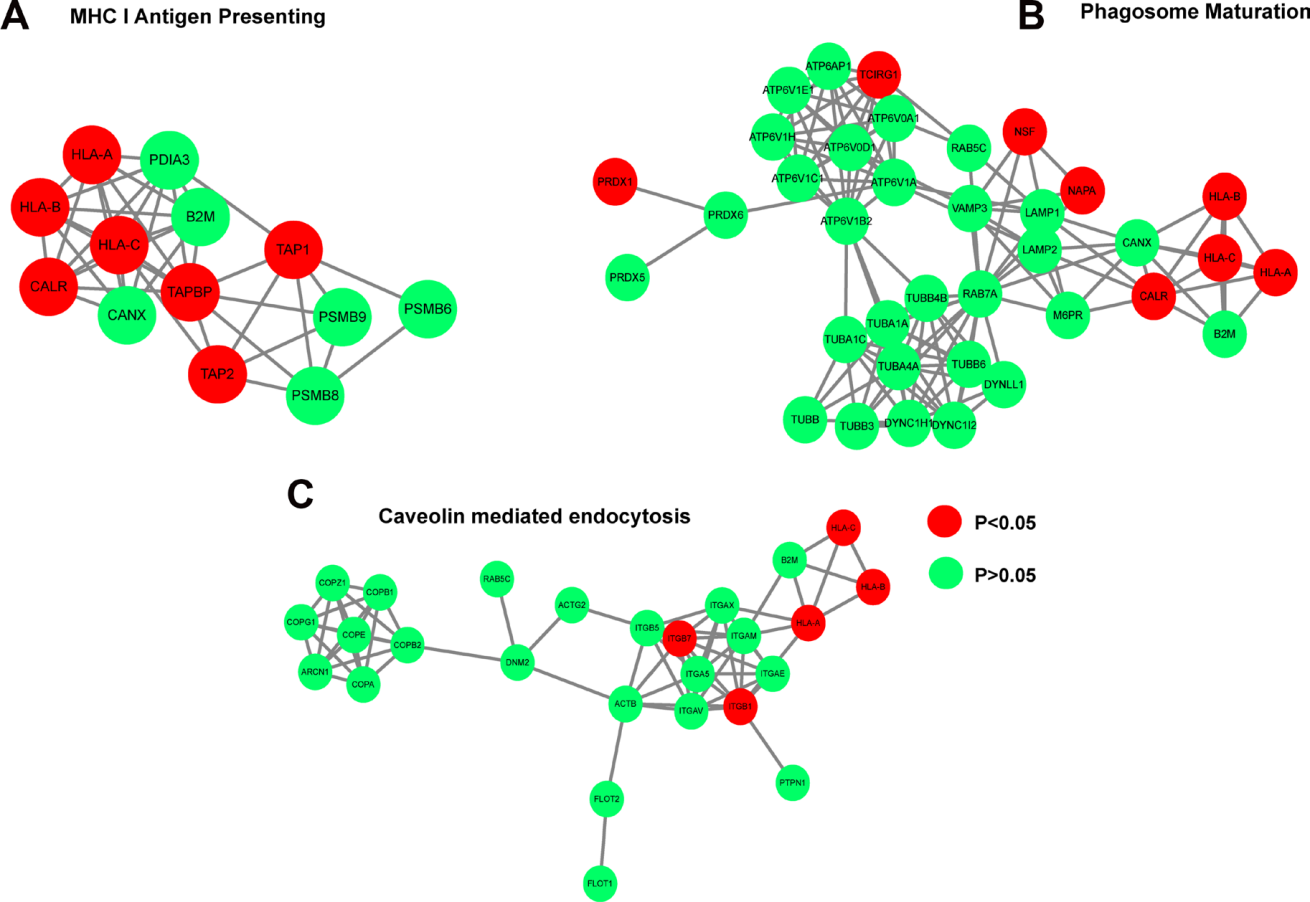
siCECR1 in THP-1 MQs enhances a protein profile associated with MHC I Antigen Presentation, Phagosome maturation, Caveolin mediated endocytosis and IFN-γ responsive signaling Networks generated by Cytoscape using IPA analysis data showing the (**A**) the MHC I presentating cluster, (**B**) Phagosome maturation cluster, (**C**) Caveolin endocytosis cluster. Nodes in red indicate proteins significantly enriched in siCECR1 MQs (*P* < 0.05). Green nodes indicate proteins present in both siSham and siCECR1 transfected MQs (n.s.). Connections indicate associations between individual proteins (either co-expression, co-localization, direct binding or pathway involvement).

Gene ontology analysis indicated that the 35 downregulated proteins were enriched in pathways including “protein folding”, “translation”, and “peptide metabolic process” (Figure 2C). The 67 siCECR1 upregulated proteins were enriched in several Gene Ontology and KEGG top-ranked pathways and intracellular mechanisms. These included “Antigen processing and presentation of exogenous peptide antigen via MHC class I” (*P* = 5.03 × 10^–5^), “type I interferon signaling pathway” (*P* = 0.0002) as well as “Phagosome” (*P* = 2.93 × 10^–6^), “Antigen processing and presentation” (*P* = 2.93 × 10^–6^) (Figure 2D and 2E). For more in-depth analysis, proteins that were non-significantly affected by CECR1 siRNA were analyzed together with the 67 upregulated proteins, using Ingenuity Pathway Analysis (IPA). Retrieved significant networks included “MHC I Antigen Presenting” (*p* < 0.0001), “Phagosome maturation” (*p* < 0.0001) and “Caveolin mediated endocytosis” (*p* < 0.0001). These three pathways are in line with our findings by GO and KEGG pathway database. Networks and detailed information of protein components of each network of are presented in Figure 3A–3C. Among the 67 proteins that were upregulated in the siCECR1 condition, 15 proteins including HLA-A, B, C, TAPBP, TAP1, ISG15, ELAVL1 and CTSS were predicated to be downstream targets of IFN-γ signaling (Supplementary Figure 2). Together these data indicate that CECR1 in MQs is involved in regulating MHC-I antigen presentation, phagosome maturation, caveolin mediated endocytosis and IFN-γ regulated signaling.

Changes in proteomic profile of U87 stimulated THP-1 macrophages in response to CECR1 silencing

**Figure 4:**
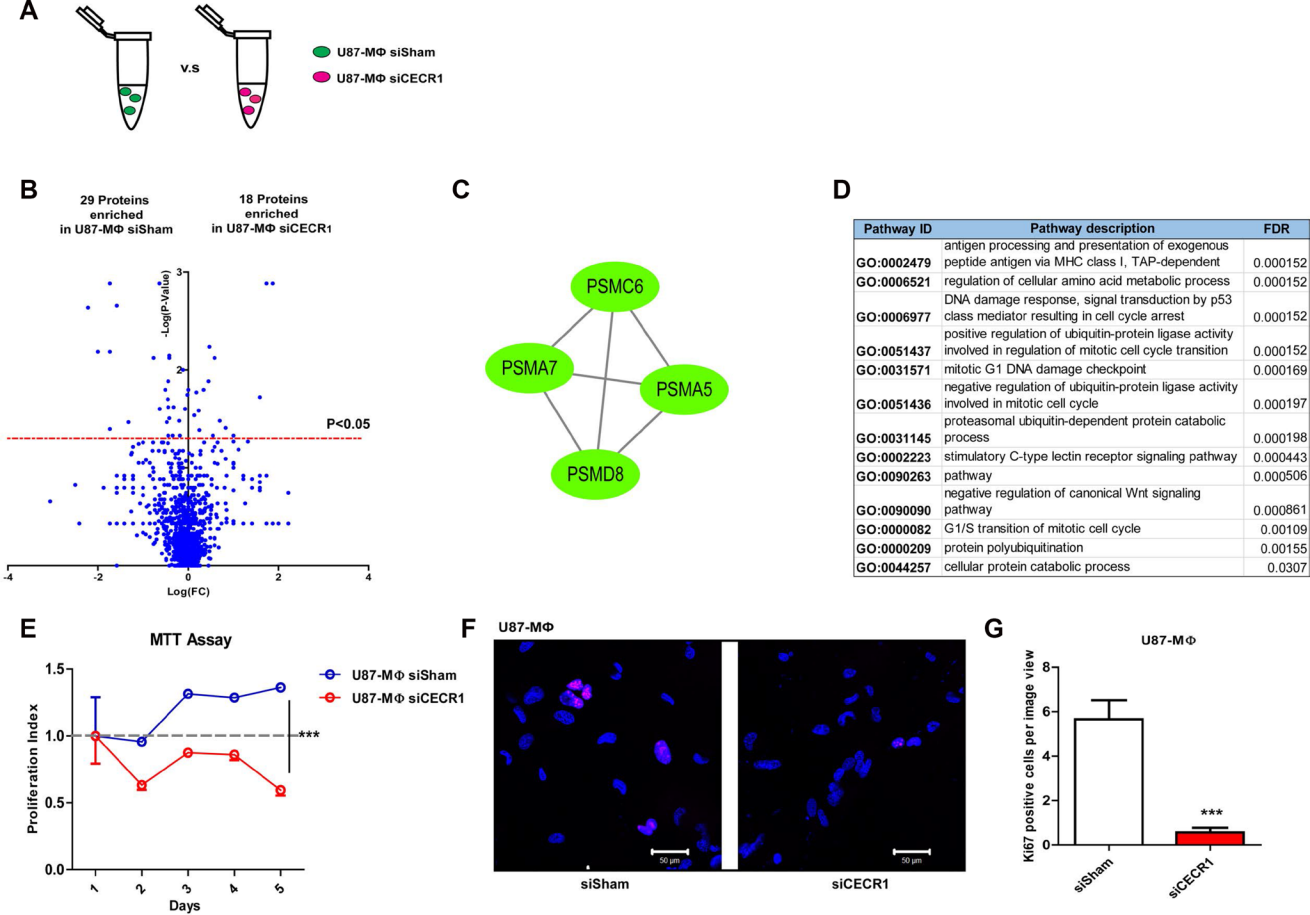
Changes in proteomic profile of U87 stimulated THP-1 MQs in response to siCECR1 (**A**) Schematic display of the comparison of siCECR1 and siSham transfected THP-1 derived MQs co-cultured with U87 cells. (**B**) Volcano blot showing differentially expressed proteins in siSham and siCECR1 transfected MQs co-cultured with U87 cells. Proteins were considered differentially expressed if *P*-value < 0.05. Expression levels of proteins are displayed on X-axis in log fold change. *P*-values are displayed on Y-axis log fold. (**C**) Network generated by Cytoscape based on STRING analysis of proteasome proteins PSMC6, PSMA5, PSMA7 and PSMD8. Green nodes indicate proteins present enriched in siSham transfected MQs in U87 co-culture condition. Connections indicate associations between individual proteins (either co-expression, co-localization, direct binding or pathway involvement). (**D**) Table listing the top 13 pathways associated with the proteasome network with FDR < 0.05. Data derived from Gene Ontology analysis. (**E**) MTT assay determining proliferation of U87 co-cultured MQs at different time points. Data are shown in mean ± SEM. Two-way ANOVA was used to test the difference of U87 co-cultured siSham MQs versus siCECR1 MQs from day 1 to day 5. ^***^P < 0.005. *N* = 3, six replications per experiment. (**F**) Ki67 staining (purple) of U87 co-cultured siSham versus siCECR1 MQs. Scale bar: 50 µm. (**G**) Quantification of Ki67 positive cells per image in U87 co-cultured MQs with siSham and siCECR1 transfection. Data shown as mean ± SEM. ^***^P < 0.005 based on *T*-test analysis. *N* = 3.

PMA-treated THP-1 cells (MQs) treated with siCECR1 were subsequently co-cultured with U87 cells to mimic TAM differentiation conditions. Results were compared with U87 co-cultured MQs treated with siSham (Figure 4A). In total, 47 proteins were significantly affected (Figure 4B); 18 proteins were upregulated by siCECR1 in co-culture and 29 proteins were downregulated (Supplementary Tables 3 and 4). Gene ontology analysis could not identify any curated pathways that were significantly annotated to the 18 upregulated proteins. The majority of pathways that were significantly linked to the 29 down-regulated proteins were based on four proteasome proteins; PSMC6, PSMA7, PSMA5, and PSMD8 (Figure 4C and 4D). The most relevant pathways were all primarily involved in cell cycle regulation (Figure 4D). In line with these findings, the proliferation rate of U87-stimulated siCECR1 MQs was lower than U87-stimulated siSham control MQs, as shown by MTT assay (Figure 4E). In addition, the number of cells positive for Ki67 (a marker for cell proliferation), in U87-stimulated siCECR1 MQs was significantly lower than in siSham control MQs (Figure 4F and 4G). Although PSMC6, PSMA7, PSMA5, and PSMD8 are involved in antigen presentation (Figure 4C and 4D) other proteins that are vital for the proteosome step in this pathway such as TAP, TAPBP and HLA-A, B, C were not enriched in siCECR1 MQs (Supplementary Figure 3), indicating that this process is not affected by CECR1 under U87 co-culture conditions.

Stimulation by U87 cells attenuates the changes that occur at protein and mRNA level in response to siCECR1 MQs

**Figure 5:**
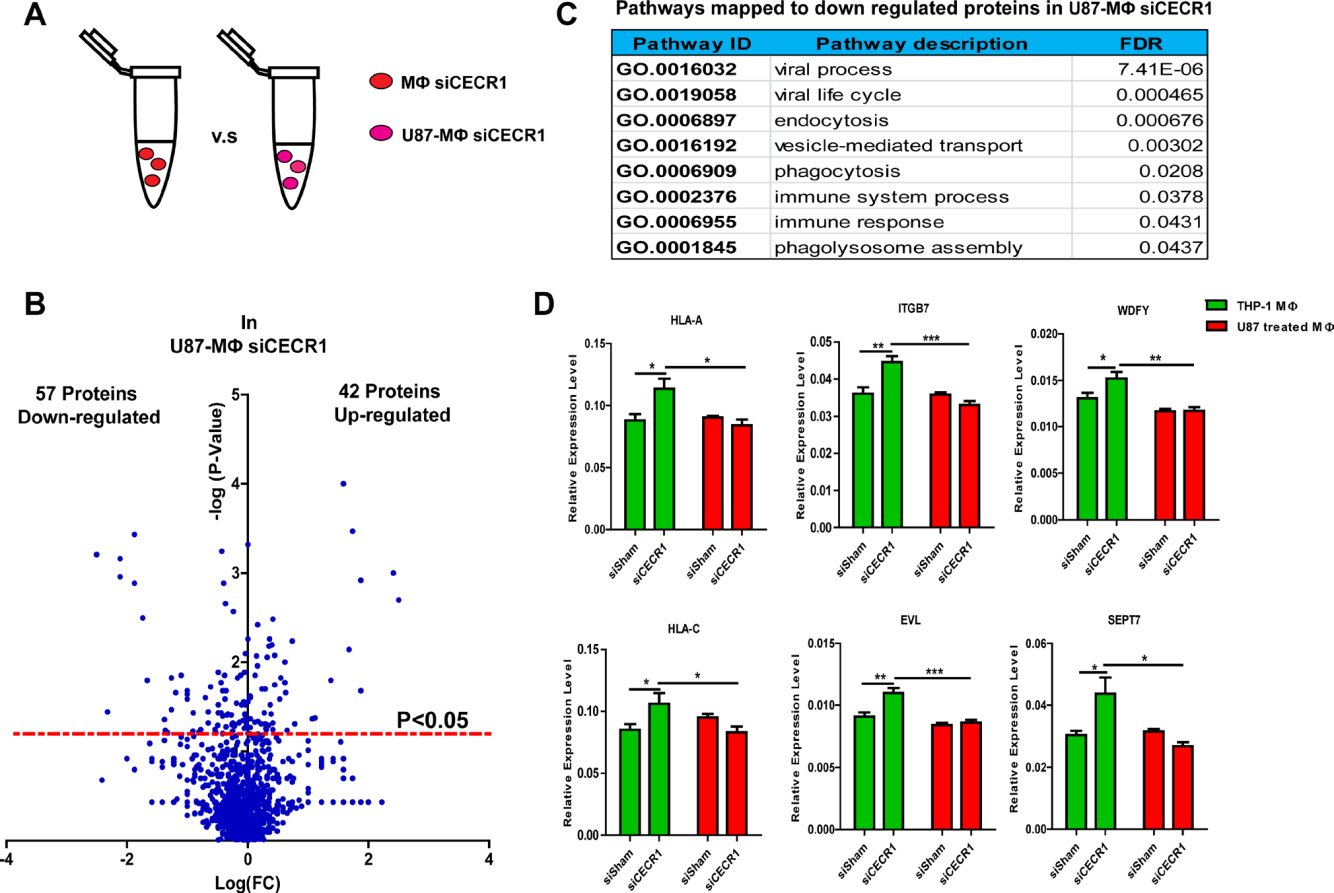
Stimulation by U87 cells attenuates the changes in MQs that occur on protein and mRNA level in response to siCECR1 (**A**) Schematic display of the comparison of siCECR1 MQs with/without co-culture of U87 cells. (**B**) Volcano blot showing differentially expressed proteins (left downregulated, right upregulated) in siCECR1 MQs co-cultured with U87 cells. Proteins were considered differentially expressed if *P*-value < 0.05. Expression levels of proteins are displayed on X-axis in log fold change. *P*-values are displayed on Y-axis log fold. (**C**) Table listing top 8 pathways with FDR < 0.05 related to downregulated proteins in U87 co-cultured MQ with CECR1 knockdown. (**D**) Real time PCR validation of HLA-A, HLA-C, ITGB7, WDFY, EVL, SEPT7 in MQs with siCECR1 and with/without U87 co-culture. Data were shown as mean ± SEM. The difference between each group was tested by student’s *T*-Test. ^*^P < 0.05, ^**^P < 0.01, ^***^P < 0.005. *N* = 3.

Only a very limited effect of siCECR1 on immune response was observed in siCECR1 MQs co-cultured with U87 cells. To investigate if the siCECR1 phenotype could be rescued by co-culturing, a comparison with or without U87 co-culture was conducted on siCECR1 MQs (Figure 5A). 57 proteins were downregulated in U87 co-cultured siCECR1 MQs compared to siCECR1 MQs without U87 stimulation (Figure 5B). The annotation of Gene Ontology analysis using the down-regulated proteins as input, indicated that “endocytosis”, “phagocytosis”, “immune response” as well as “phagolysosome assembly” may be affected (Figure 5C). To assess whether mRNA levels were equally downregulated, transcripts of key molecules involved in pathways of Figure 5C, like HLA-A, C, ITGB7, EVL, WDFY, SEPT7 were evaluated in MQs with/without U87 co-culture. As shown, SiCECR1 increased gene expression of these molecules. However, U87 co-culture attenuated this upregulation of target genes in response to siCECR1 (Figure 5D). Thus, the changes observed on protein level in response to siCECR1 in MQs are regulated on mRNA gene expression level, but U87 stimulation diminishes this response.

Overlap analysis identifies S100A9 and PLAU as two CECR1-related proteins that are significantly correlated with expression of CECR1 and MQ lineage markers in three large-sized public GBM datasets

**Figure 6:**
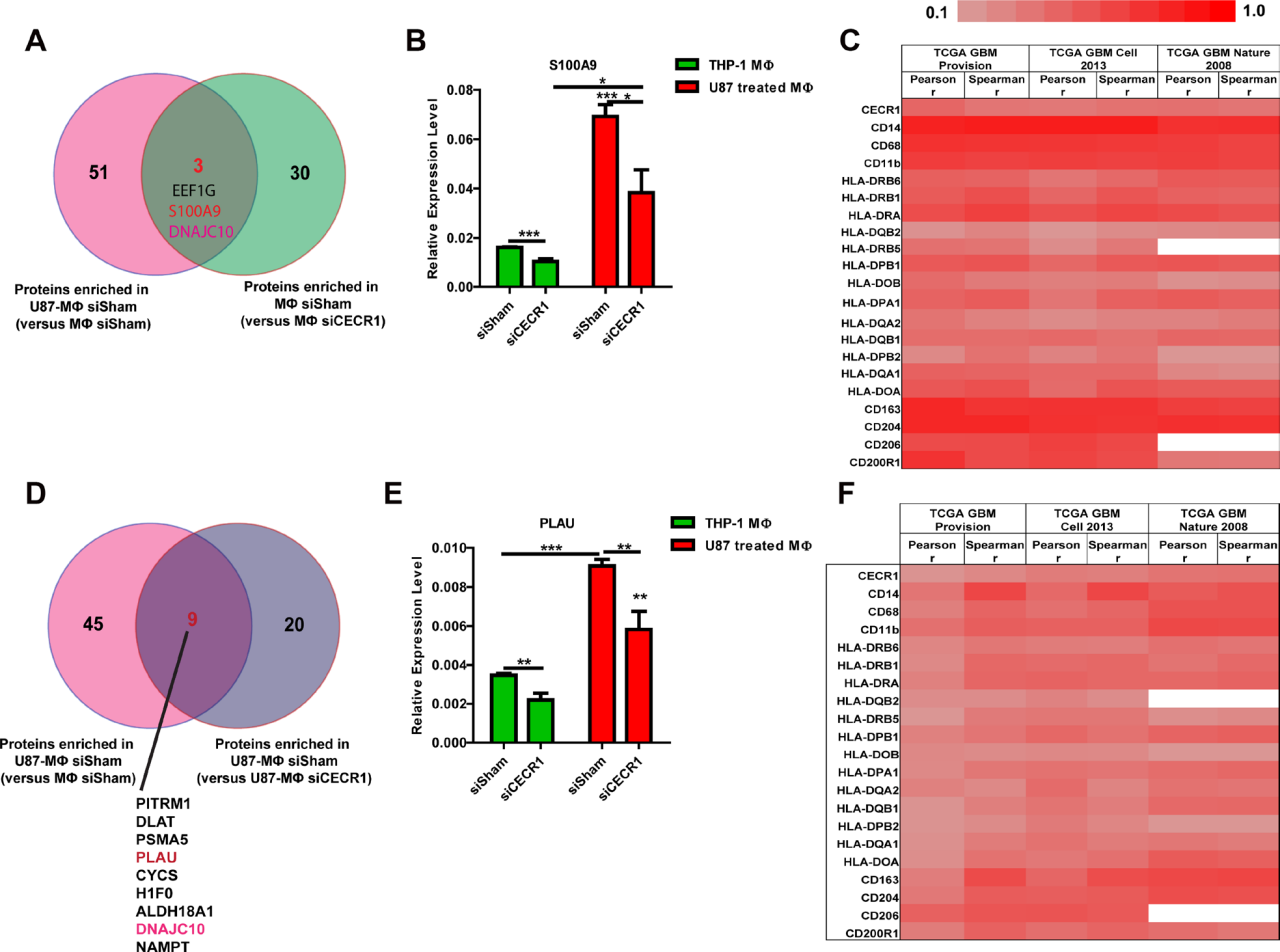
Overlap analysis identifies S100A9 and PLAU as two proteins significantly correlated with expression of CECR1 and MQ lineage markers in three large public GBM datasets (**A**) Venn graph showing the overlapping proteins from proteins enriched in U87 co-cultured siSham MQs (versus siSham MQs without U87 stimulation) and proteins enriched in siSham MQs (versus siCECR1 MQs). (**B**) Real time PCR validation of S100A9 expression in siCECR1 MQs with/without U87 co-culture. Expression levels are displayed relative to housekeeping gene. The difference between each group was tested by student’s *T*-Test. Data are shown as mean ± SEM. ^*^P < 0.05, ^***^P < 0.005. *N* = 3. (**C**) Heat map showing correlation co-efficiency from both Pearson and Spearman analysis of S100A9 with CECR1 and monocyte/macrophage markers in three TCGA GBM datasets. (**D**) Venn graph showing the overlapping proteins from proteins enriched in U87 co-cultured siSham MQs (versus siSham MQs without U87 stimulation) and proteins enriched in U87 co-cultured siSham MQs (versus siCECR1 MQs). (**E**) Real time PCR validation of PLAU expression in siCECR1 MQs and with/without U87 co-culture. Expression levels are displayed relative to housekeeping gene. The differences between groups were tested by student’s *T*-Test. Data are shown as mean ± SEM. ^**^P < 0.01, ^***^P < 0.005; *N* = 3. (**F**) Heat map showing correlation co-efficiency from both Pearson and Spearman analysis of PLAU with CECR1 and monocyte/macrophage markers in three TCGA GBM datasets.

As shown in our previous study, CECR1 is highly expressed in MQs stimulated with conditioned medium of U87 cells [23]. MQ-derived proteins may thus be putatively regulated by CECR1. Next, we compared the list of proteins that were upregulated in U87-stimulated MQs versus MQs without U87 treatment (Supplementary Table 5) with the list of proteins that were downregulated in siCECR1 MQs compared to siSham MQs (Supplementary Table 1). This overlap analysis retrieved three common proteins; EEF1G, S100A9 and DNAJC10 (Figure 6A). Similarly, the comparison of enriched proteins in U87-stimulated MQs versus non-stimulated MQs (Supplementary Table 6) with downregulated proteins in U87 co-cultured siCECR1 MQs versus U87 co-cultured siSham MQs (Supplementary Table 4) resulted in 9 common proteins (Figure 6D). QPCR analysis validated the downregulation of S100A9 and PLAU expression in siCECR1 MQs with/without U87 co-culture compared to their siSham controls. U87 stimulation significantly increased expression of S100A9 and PLAU in MQs (Figure 6B and 6E). Analysis of S100A9 and PLAU in the TCGA GBM database using 3 large-size public GBM datasets demonstrated that both S100A9 and PLAU expression levels are positively correlated with CECR1 and other monocyte/macrophage lineage markers (Figure 6C and 6F). Although the overlap analyses indicated that DNAJC10 is the most relevant molecule under regulation of CECR1, the analysis of DNAJC10 using the same public GBM datasets did not show any correlation with CECR1 as well as other markers of monocytes/macrophages (data not shown).

### LAT2 was up-regulated by siCECR1 in U87 co-cultured MQs

**Figure 7:**
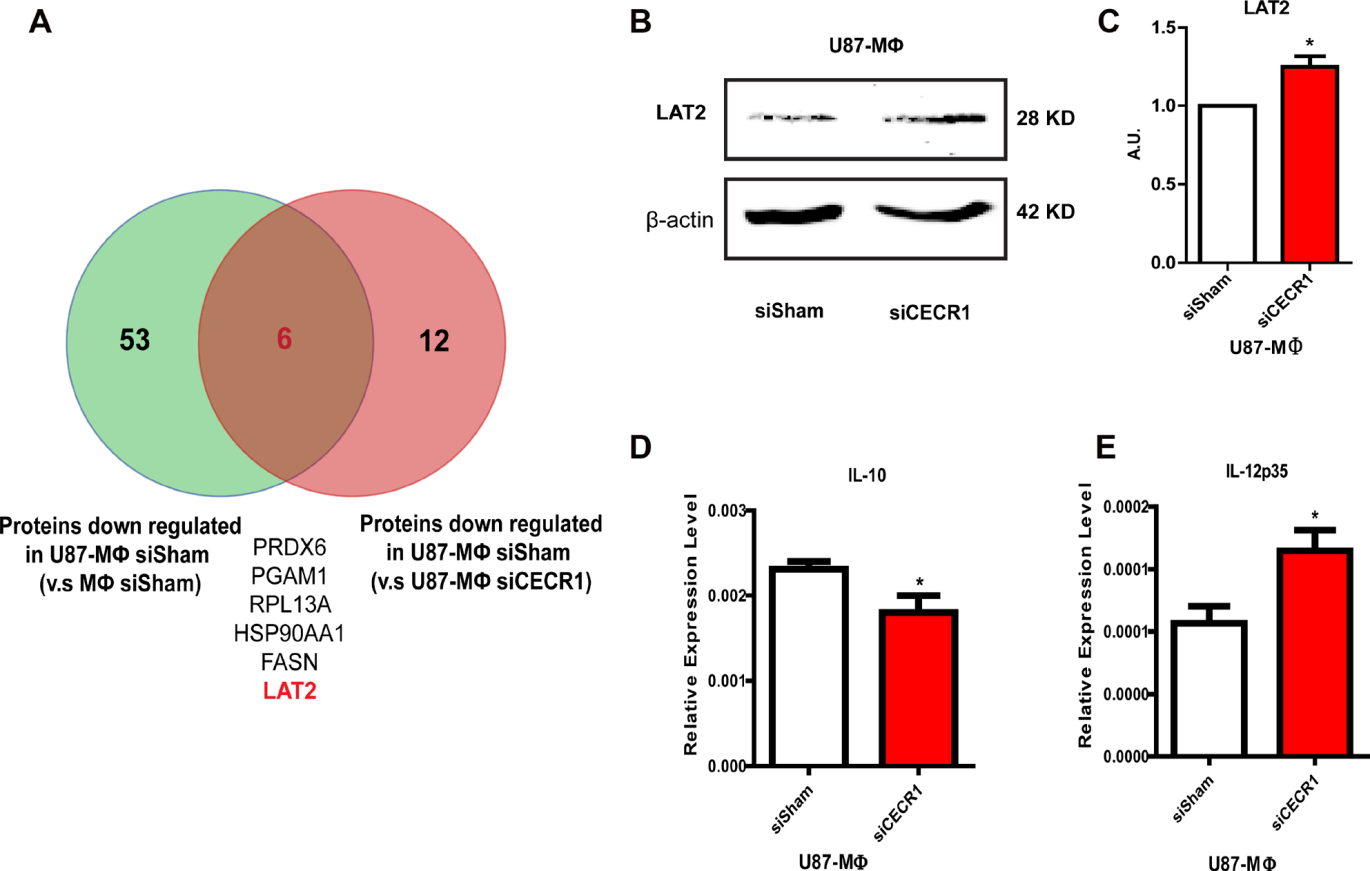
LAT2 is upregulated by siCECR1 in U87 co-cultured macrophages (**A**) Venn graph showing overlapping proteins from proteins enriched in U87 co-cultured siSham MQs (versus siSham MQs without U87 stimulation) and proteins enriched in siSham MQs with U87 (versus siCECR1 MQs with U87). (**B**) Representative Western blot of LAT2 protein in U87 co-cultured macrophages with siSham and siCECR1 transfection. (**C**) Quantification of LAT2 protein in U87 co-cultured MQs with siSham and siCECR1. Protein signal was corrected for loading control (β actin). Data shown as mean ± SEM. ^*^P < 0.05 based on *T*-test analysis. *N* = 3. (**D**) Real time PCR measurement of IL-10 expression in U87 co-cultured macrophages with/without CECR1 silencing. Data were shown as mean ± SEM. ^*^P < 0.05 based on *T*-test analysis. *N* = 3. (**E**) Real time PCR measurement of IL-12p35 expression in U87 co-cultured macrophages with/without CECR1 silencing. Data were shown as mean ± SEM. ^*^P < 0.05 based on *T*-test analysis. *N* = 3.

**Figure 8:**
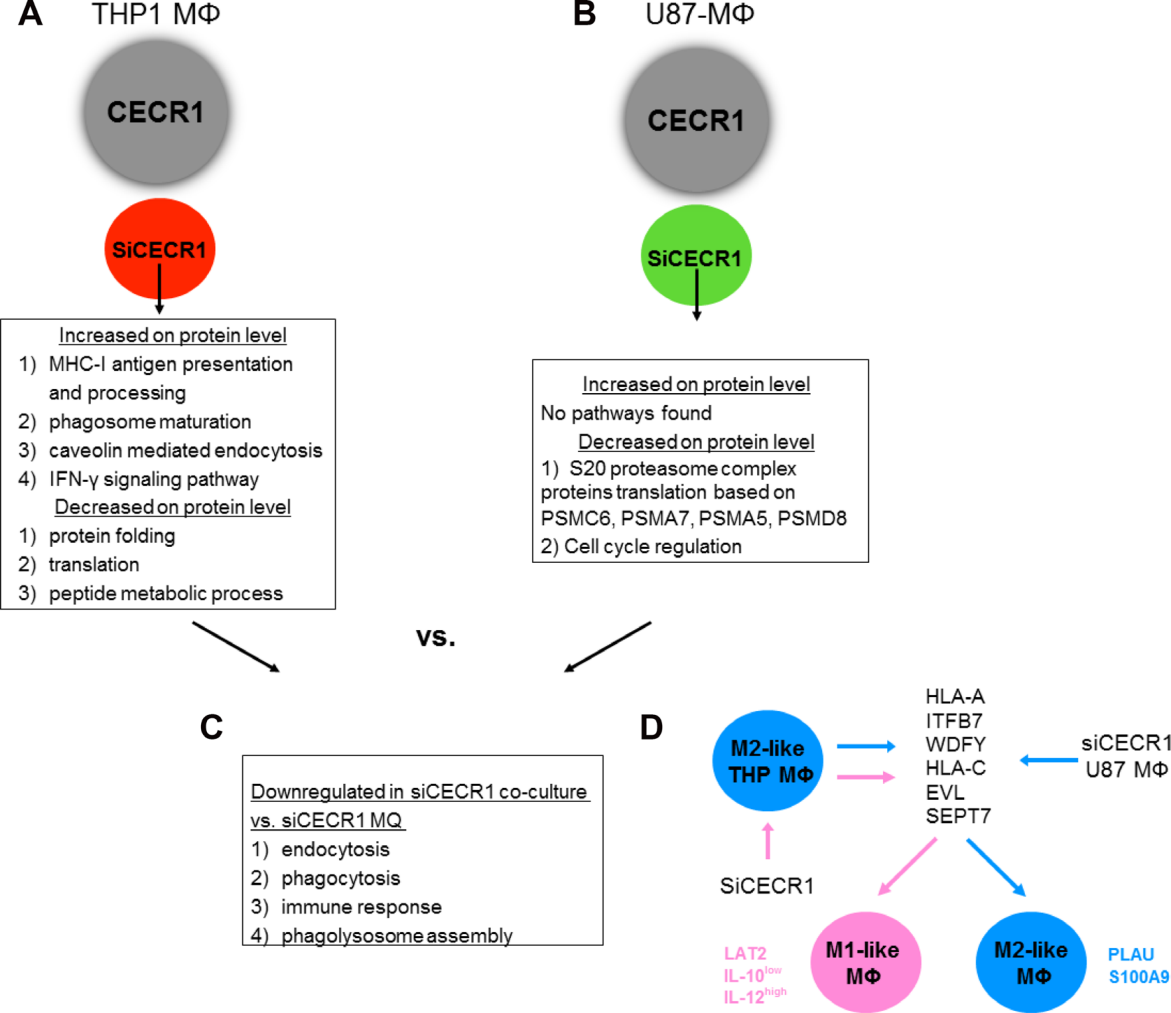
Schematic representation of siRNA-associated immunostimulation (**A**) SiRNA treatment of CECR1 on isolated THP-1 macrophages (MΦ) results in an increase of MHC-I antigen processing and IFN-γ signaling pathways, resulting in an M1-like phenotype. (**B**) SiRNA treatment of CECR1 on co-cultured MΦ and U87-glioma cells results in an decrease of pathways associated with proteasome and cell cycle regulation, leading to lower MΦ proliferation rates. (**C**) Co-culture with U87-glioma cells compared to siCECR1 MΦ without U87-glioma cells results in decrease in protein and RNA expression of targets associated with immune response and phagocytosis. (**D**) CECR1 normally regulates genexpression of several proteins and pathways associated with MHC-I antigen processing and IFN-γ signaling, resulting in an M2-like phenotype. SiCECR leads to increased expression of these targets, leading to a more M1-like phenotype. Several M2-MΦ lineage markers, e.g. PLAU and S100A9, are upregulated by U87 stimulation. However, LAT2 expression combined with an IL-10 low and IL-12p35 high profile are still present in siCECR1-U87 stimulated cells.

Further overlap analysis identified Linker For Activation Of T Cells Family Member 2 (LAT2) as a CECR1-related protein (Figure 7A) via comparison of downregulated proteins in U87-stimulated versus non-stimulated MQs (Supplementary Table 6) with proteins upregulated in U87 co-cultured siCECR1 MQs versus siSham MQs (Supplementary Table 3). LAT2 was upregulated as shown by Western Blot in U87 co-cultured siCECR1 MQs (Figure 7B and 7C). In line with this finding, U87 co-cultured siCECR1 MQs displayed an IL-10 low and IL-12p35 high expression profile, which implied a more pro-inflammatory (M1) phenotype (Figure 7D and 7E). A schematic overview of our major findings is depicted in Figure 8.

## DISCUSSION

Recent studies have highlighted the importance of CECR1 in the regulation of the innate immune response [20]. *In vitro*, monocytes derived from patients with deleterious CECR1 mutations showed diminished capacity of M2 macrophage differentiation, whereas M1 macrophage differentiation remained unaffected [21]. In glial tumors, the TAM population mainly exhibits a M2-MQ like phenotype. We previously reported that CECR1 was strongly expressed by M2-like TAMs in high-grade gliomas. In line with the results of the genetic study, CECR1 proved vital for differentiation of monocytes towards M2-like TAMs in a glial tumor paracrine environment by means of U87 conditioned medium stimulation [23]. CECR1 activity in M2-like TAMs also promoted paracrine stimulation of glial tumor cell migration and proliferation [23]. In addition, CECR1 function facilitated paracrine crosstalk of M2-like TAMs with perivascular pericytes, contributing to tumor angiogenesis [24].

Although these studies provide direct evidence for the significant role of CECR1 in TAM regulation, the molecular mechanisms that drive the CECR1-mediated function of TAMs remains to be elucidated. A genome-wide “omics” approach could help reveal CECR1 mechanisms. A previously published genome-wide transcriptome dataset of peripheral blood mononuclear cells (PBMCs) derived from patients with CECR1 mutations, showed marked increased expression of neutrophil-associated genes in disease condition [25]. Another case-report study did not detect upregulation of neutrophil-associated genes in the patient’s PBMCs, but demonstrated an increase in IFN-γ response genes [26]. In line with this report, patients with CECR1 genetic mutations that received anti-TNF-α antibody treatment reached complete remission or partially recovered from their autoimmune disease-like symptoms [27]. The latter clearly demonstrates the potential value of in depth analysis of CECR1 mediated mechanisms in identifying new drug targets for therapy development. As most molecular data on CECR1 function in immune cells were obtained from the mixed PBMC population, these findings remain difficult to extrapolate to aid in our understanding of the CECR1 working mechanism of local TAMs in glial tumors. The general working mechanism of CECR1 in monocytes/macrophages also requires further exploration.

In this study, we conducted proteomic analysis of siRNA mediated CECR1 silencing in THP-1-derived MQs that were stimulated with or without glial tumor cells (U87) in co-culture. We validated siCECR1 efficiency and selectivity by employing 2 separate siRNA systems. As a second validation, we confirmed increased RNA expression of the 5 targets with the highest fold change found in the proteomics analysis (MQ siCECR1 vs. MQ siSham), using the siCECR1 system from Sigma Aldrich.

In the absence of U87 stimulation, siCECR1 in MQs showed a protein profile related to key immune response regulation mechanisms in MQ, including “MHC I antigen presenting”, “Phagosome maturation”, “Caveolin mediated endocytosis” and “Type I interferon signaling pathway”. These pathways are mostly involved in pro-inflammatory responses and anti-pathogen defense, and are more in line with M1-macrophage function. Among these three pathways, “MHC I Antigen Presenting” and “Phagosome maturation” are also regulated via IFN-γ signaling [28]. IFN-γ has been recognized as the main cytokine that is associated to induction of the M1 response [7]. Our data showed upregulation of proteins mapping into interferon responding pathway in response to siCECR-1 of MQs, including ISG15, HLA-A, HLA-B, HLA-C, TAP1, TAP2, TAPBP, and TIMP-1. Furthermore, several proteins that were recently reported to be involved in immune response like WDFY1 and SEPT7, involved in phagosome assembly and vesicle transport [28], were also enriched in CECR1-silenced MQs. WDFY1 is an adaptor protein anchored on the membrane of early endosomes. It participates in Toll like receptor pathways and activates NF-κB signaling pathway in MQs, which in turn, activates the transcription of type I interferons. Other activated inflammatory factors include key proteins of the M1 macrophage cytokine release repertoire such as TNF-α, IL-6 and CXCL10 [29, 30]. Moreover, siCECR1 in MQs promotes higher levels of proteins associated with the MHC I antigen presenting function. This would imply enhanced CD8+ T cell activation, a feature that is associated with M1-macrophages [31]. Thus, in line with present literature and our previous studies, current data shows that CECR1 function in MQs is indeed important to endorse M2 macrophage function, made evident by the shift towards a more M1 macrophage-like proteosome profile observed in response to siCECR1 treatment.

Malignant tumors such as glial tumors are known to influence the local immune response, including hampering vital MQ functions like antigen presenting and phagocytosis [32]. In this study, we observed a general inhibitory effect of U87 cell on the CECR1-silencing response in MQs: co-culture with U87 cells reversed the siCECR1 induced pro-inflammatory M1-like protein profile in MQs. No upregulation of proteins involved in the “MHC I antigen presenting”, “Phagosome maturation”, “Caveolin mediated endocytosis” and “Type I interferon signaling pathway” were observed. In contrast, siCECR1 in MQs under U87 co-culture mainly downregulated proteins part of the cell cycle regulation. In line with this proteome profile, siCECR1 MQs in U87 co-culture indeed showed a lower proliferation capacity compared to siSham treated controls. MQ proliferation was reportedly activated by paracrine stimulation of malignant tumor cells, mainly via secretion of various cytokines and growth factors like M-CSF [33, 34]. Numerous studies have indicated that reduction of the number of immuno-suppressive (M2) MQs could be an effective anti-tumor therapeutic approach [34, 35]. Our proteomic analysis revealed downregulation of the proteasome proteins PSMA5, PSMA7, PSMC6 and PSMD8 in siCECR1 U87 co-culture conditions. PSMA5 and PSMA7 are essential subunits of the complete assembly of the 20S proteasome core complex, whereas PSMC6 and PSMD8 are essential subunits of the 19S regulatory particle. Together, 1 20S core and 2 19S regulator particles form the functional 26S proteasome complex [36]. The 26-proteasome complex participates in various critical cell pathways including DNA synthesis, repair, transcription, translation and cell signal transduction [36] as well as antigen presentation [37] and regulation of cell cycle transition [38]. Cell proliferation and the cell cycle are mainly regulated by 26S proteasomes by targeting p53 [39] and inhibitors of cell cycle dependent kinase, like p21 [40], p27 [41], for ubiquitin-mediated degradation. Inhibition of 26S proteasome activity led to cell cycle arrest and induced apoptosis by increasing levels of p53, p21 and p27 [42]. Our findings indicate that CECR1 silencing may contribute to low proliferation rate of TAMs via downregulation of essential protein components of the S26 proteasome complex. Thus, CECR1 depletion may be considered as a novel approach in targeting TAM proliferation in gliomas.

Overlap analysis of our data identified S100A9 and PLAU as two CECR1-related proteins that are significantly correlated with expression of CECR1 and MQ lineage markers in GBMs. These two molecules are highly expressed in myeloid cells [10, 43]. S100A9 was also shown to promote glioma growth and angiogenesis by interaction with the RAGE receptor on the surface of glioma cells [44], a process mediated via activation of MAPK and NF-κB pathways [45]. PLAU is one of the pro-angiogenic factors that can be produced by TAMs [10]. It was shown to aid the invasion and metastasis of various types of cancer cells via binding to PLAUR, subsequently activating downstream pathways like ERK1/2 [46] and PI3K/Akt [47]. Moreover, the interaction of PLAU-PLAUR raised plasmin levels, contributing to the activation of matrix metalloproteinases [48].

In U87 co-cultured MQs, siCECR1 upregulated protein levels of LAT2. Coincidently, U87 co-culture decreased the LAT2 expression in siSham MQs. Previous studies pointed to an immune modulatory function of LAT2 via competing with TREM-2/LAT signaling pathway, which resulted in upregulation of IL-12 and downregulation of IL-10 in LPS stimulated MQs [49]. Indeed, siCECR1 MQs in U87 co-culture conditions displayed a decrease in their IL-10/IL-12p35 ratio.

In conclusion, in this study we have identified, for the first time to our knowledge, the molecular pathways and key molecules that are mediated by CECR1 function in MQs and glial TAMs. Our proteome dataset could provide the basis for the development of interesting drug targets for future immunotherapy development in the treatment of malignant (glial) tumors as well as auto immune disease.

## MATERIALS AND METHODS

### Cell cultures

The human monocytic cell line, THP-1 cells were obtained from the department of Hematology, Erasmus Medical Center, Rotterdam. THP-1 cells were cultured in RPMI-1640 medium (Cat:BE12-702F/U1, Lonza, Breda, The Netherlands) supplemented with 10% fetal bovine serum (FBS) (Cat: 10500064, Thermo Fisher Scientific, Bleiswijk, the Netherlands) and 1% Penicillin/Streptomycin (Cat:15140-122, Thermo Fisher Scientific). THP-1 macrophage differentiation was induced by stimulating with PMA (P8139, Sigma-Aldrich, Zwijndrecht, the Netherlands) for 48 hours at a concentration of 100 ng/ml. The human GBM cell line U87 was purchased from ATCC (USA) and maintained in DMEM (Cat: 12-604F, Lonza, Breda, The Netherlands) with 10% FBS and 1% Penicillin/Streptomycin.

### SiRNA transfections

A mix of four siRNA sequences that target CECR1 mRNA (5′-GUGCCAAAGGCUUGUCCUA-3′, 5-CUUCCACGCCGGAGAAACA-3′, 5′-GCCCAAAGCUAGUUAGUAC-3′, 5′-UCGCAGAAUCCAUCCGAAU-3′) (Cat: L-009521-01-0005, Dharmacon, Lafayette, USA) and scrambled non-targeting siRNAs (5′-UGGUUUACAUGUCGACUAA-3′, 5′-UGGUUUACAUGUUGUGUGA-3′, 5′-UGGUUUACAUGUUUUCUGA-3′, UGGUUUACAUGUUUUCCUA-3′) were obtained from Dharmacon (Cat: # D-001810-10-20, Dharmacon, Lafayette, USA). THP-1 derived MQ cultures were transfected after 48 hours of PMA treatment, following the manufacture’s protocol (using Dharmacon transfection reagent). Efficiency of CECR1 knockdown was assessed after 24 and 48 hours at transcriptional level and protein level. For co-culture experiments, siRNA transfected cells were used 24 hours post transfection. In a second validation experiment, a mix of two siRNA sequences that target CECR1 mRNA (SASI_Hs01_00039762, SASI_Hs01_00096471), transfection reagent (MISSION^®^ siRNA Transfection Reagent, S145) and a non-targeting siRNA (MISSION^®^ siRNA Fluorescent Universal Negative Control, SIC007) were used (All Sigma-Aldrich, St. Louis, MO, USA).

### Cell co-cultures

100,000 U87 cells were seeded on top of the culture inserts with a pore size of 0.4 µm (Cat: 140640, Thermo Fisher Scientific, USA). SiRNA transfected MQs were seeded into the lower chamber in 6-well plates. MQs were co-cultured with U87 cells in RPMI-1640 medium with 1% FCS for 48 hours and 96 hours for RNA and protein isolation respectively. Real-time qPCR and proteomic analysis were performed after 48 hours and 96 hours post co-culturing respectively. MQs without U87 cell co-culture were harvested 48 hours and 96 hours post transfection for qPCR and proteomic measurement respectively (Figure 1).

### Sample preparation

MQ cell pellets were harvested and 150 µl of RapiGest (Waters Corporation, Milford, MA) in 50 mM NH4HCO3 was added to each sample. Samples were sonificated for 4 min at 70% amplitude at a maximum temperature of 25° C (Branson, Ultrasonic, Danbury, CT). The proteins were reduced with 10 mM dithiothreitol (DTT) at 60° C for 30 min. After the mixture was cooled down to room temperature, it was alkylated in the dark with 50 mM iodoacetamide at ambient temperature for 30 min, and digested overnight with 5 µl trypsin (Promega, Madison, WI). To inactivate trypsin and to degrade the RapiGest, 2 µl of 50% TFA was added and samples were subsequently incubated for 30 minutes at 37° C. Samples were centrifuged at maximum speed for 15 min at 4° C and the supernatants were transferred to LC vials to be measured on the LC-MS. The digested samples were measured on a nano-LC (Thermo Fisher Scientific, Germering, Germany).

### LC Orbitrap MS

LC-MS measurements were carried out on a nano LC system (Ultimate 3000 RSLC nano system; Thermo Fisher Scientific, Germering, Germany) online coupled to a hybrid linear ion trap/Orbitrap MS (LTQ Orbitrap XL; Thermo Fisher Scientific, Bremen, Germany). One microliters of digest or 5 µL of spiked sample was injected onto the nano LC system, which held a C18 trap column (PepMap C18, 300 μm ID × 5 mm, 5 μm particle size and 100 Å pore size; Thermo Fisher Scientific) and a C18 analytic column (PepMap C18, 75 μm ID × 500 mm, 2 μm particle size and 100 Å pore size; Thermo Fisher Scientific). A 180-minute gradient with a 250 nL/min flow was run with solvent A (H2O/acetonitrile (ACN) 98/2 (v/v), 0.1% formic acid (FA)) and solvent B (H2O/ACN 20/80 (v/v), 0.1% FA): 0–38% solvent B in 180 min. All solvents used were purchased from Biosolve (Valkenswaard, Netherlands). The separation of the peptides was monitored by a UV detector (absorption at 214 nm). High-resolution full scan MS was obtained from the Orbitrap (resolution 30,000; AGC 1,000,000), MS/MS spectra were obtained by CAD fragmentation using a NCE value 35. MS/MS was performed on the top five masses in the full scan spectra using an AGC 1,000. Dynamic exclusion was used, with a repeat count of one; exclusion duration was set at 3 min and exclusion width at ± 5 ppm.

### Data analyses

From the data files the MS/MS spectra were extracted and converted into mgf files by using MSConvert of ProteoWizard (version 3.0.06245). All mgf files were analyzed using Mascot (version 2.3.02; the Matrix Science, London, UK). Mascot was used to perform database searches against the human subset the uniprot_sprot_2015–10 database; Homo sapiens species restriction; 20,194 sequences) of the extracted MS/MS data. For the database search the following settings were used: a maximum of two miss cleavages, oxidation as a variable modification of methionine, carbamidomethylation as a fixed modification of cysteine and trypsin was set as enzyme. A peptide mass tolerance of 10 ppm and a fragment mass tolerance of 0.5 Da were allowed. Scaffold software (version 4.4.3, Portland, OR) was used to summarize and filter MS/MS based peptides and protein identifications at an FDR of 1% and contained at least one identified peptide. Proteins that contained similar peptides and could not be differentiated based on MS/MS analysis alone were grouped. From Scaffold the spectral counts per sample of all the identified proteins were exported to Microsoft Office Excel. Total unique peptide count per peptide in each group was compared by student’s *T*-Test. Proteins were considered differentially expressed when *P* < 0.05.

### Bioinformatics

Raw data generated by Scaffold 4 was exported into Excel. The dot-plots (Log2 FC vs. –log10*P*-Value) also called volcano blots were made by Prism 6.0. Significantly expressed proteins were enriched in Gene Ontology (GO), biological process pathways and KEGG pathways by using STRING database (http://string-db.org/). For validating pathways explored in Gene Ontology and KEGG pathway database, Ingenuity Pathway Analysis (Qiagen, USA) was applied. For network visualization, firstly, proteins that were not significantly changed were imported as background molecules and mapped into canonical pathways together with significantly expressed proteins. Proteins mapped into Canonical Pathways (FDR < 0.05) were exported, and networks were generated by STRING online software. Information of each network were exported and visualized using Cytoscape 3.3.

Mutual expressed proteins were identified and visualized by Venny Graphs (http://bioinfogp.cnb.csic.es/tools/venny/). Correlation analysis was performed using the TCGA GBM database via the c-Bioportal provided by the MSKC Center. Calculated Pearson and Spearman correlation co-efficiencies were used to generate Heat maps using Excel.

### RNA isolation and Real time qPCR

Total RNA was isolated from MQs using RNA isolation kit (BIO-52073, Bio-line, USA) and reversely synthesized into cDNA using Sensi-fast cDNA synthesis kit (BIO-65053, Bio-line, USA) following manufacture’s protocol. QPCR was performed by assessment of SYBRGreen signal using CFX96 Touch™ Real-Time PCR Detection System (Bio-rad, USA). Transcripts of CECR1, S100A9, PLAU, CTSH, HLA-A, HLA-C, ITGB7, WDFY, EVL, GALM, SEPT7, IL-10, IL-12p35 were measured and normalized to β-actin (Primers for all targets are shown in Supplementary Table 7).

### MTT assay

PMA-treated THP-1 MQs were transfected with siSham and siCECR1. The following day, transfected MQs were seeded in 96-well plates at a density of 5000 cells/well. MQs were treated with U87 derived conditioned medium (CM). MQs without U87 CM treatment were measured at the same day as a reference measurement. MQ proliferation was monitored for the next five days using MTT, 3-(4,5-dimethylthiazol-2-yl)-2,5-diphenyltetrazolium bromide (Cat:M5655, Sigma-Aldrich, the Netherlands), which was added at a concentration of 0.45 mg/ml to each well. After 4 hours of incubation, the MTT signals were measured by spectrometry (Thermo Fisher Scientific) at the 570 nm absorbance wavelength.

### Western blotting

20 µg of total protein was loaded onto a 10% SDS-PAGE gel (Cat: #1610158, Bio-Rad, USA) and blotted to Nitro cellulose membranes (Cat: 88018, Thermo Fisher Scientific, the Netherlands) followed by incubation with block buffer (Cat: P/N 927-40000, Licor, Bioscience, USA). Protein levels were assessed by immunoblotting using specific antibodies against LAT2 (Cat: 11986S, Cell Signaling Technology, Leiden, the Netherlands, 1:1000), and β-actin (Cat: ab8229, Abcam, Cambridge, UK, 1:500) as a loading control, followed by incubation with secondary antibodies (IRdye 680 CW, Cat: P/N 925-68071, P/N 925-68074; IRDye 800 CW, Cat: P/N 925-32211, P/N 925-32214, Licor Bioscience, USA) and detection of signals using the Odyssey imaging system (Licor Bioscience, USA).

### Immunofluorescent staining

SiCECR1 MQs versus siSham-transfected controls were treated with U87 CM for 48 hours. MQs were fixed in 4% PFA for 15 min. Cells were permeabilized followed by a blocking step. Primary antibodies against CECR1 (Cat: HPA007888, Sigma-Aldrich, the Netherlands, 1:200), Ki67 (Cat: M7240, Dako, Denmark, 1:200), and secondary antibodies of Goat Anti-Rabbit Alexa Fluor 488 (Cat: A-11008, Thermo Fisher Scientific, the Netherlands, 1:200) and anti-Mouse Alexa Fluor 555 (Cat: A-21422, Thermo Fisher Scientific, the Netherlands, 1:200) were applied to detect expression of target proteins. 10 areas were randomly selected and pictures were taken under the confocal microscope (LSM-700, Zeiss, The Netherlands). The number of Ki67 positive cells was counted in each image view.

### Statistics

Unique peptides from the proteomic analysis were compared per group in each experiment and analyzed using unpaired two-tailed students’ *T*-Test. Proteins were considered significantly differentially expressed when *P* < 0.05. All *in vitro* data were tested using unpaired two-tailed student’s *T* test (Significance levels *P* < 0.05). All data are presented in Mean ± S.E.M. unless otherwise stated.

## SUPPLEMENTARY MATERIALS FIGURES AND TABLES









## Abbreviations

TAM: tumor associated macrophage
CECR1: Cat Eye Syndrome Critical Region Protein 1
PMA: phorbol myristate acetate
MQ: macrophage
GBM: glioblastoma multiforme
