# Supplementary material for: Comparative proteomic analysis of cat eye syndrome critical region protein 1- function in tumor-associated macrophages and immune response regulation of glial tumors
Source: Oncotarget. 2018 Sep 11;9(71):33500–14. doi: 10.18632/oncotarget.26063 (PMC6173361; doi:10.18632/oncotarget.26063)
Supplement: Supplementary file 1 [file oncotarget-09-33500-s001.pdf]

# Comparative proteomic analysis of cat eye syndrome critical region protein 1- function in tumor-associated macrophages and immune response regulation of glial tumors

## SUPPLEMENTARY MATERIALS

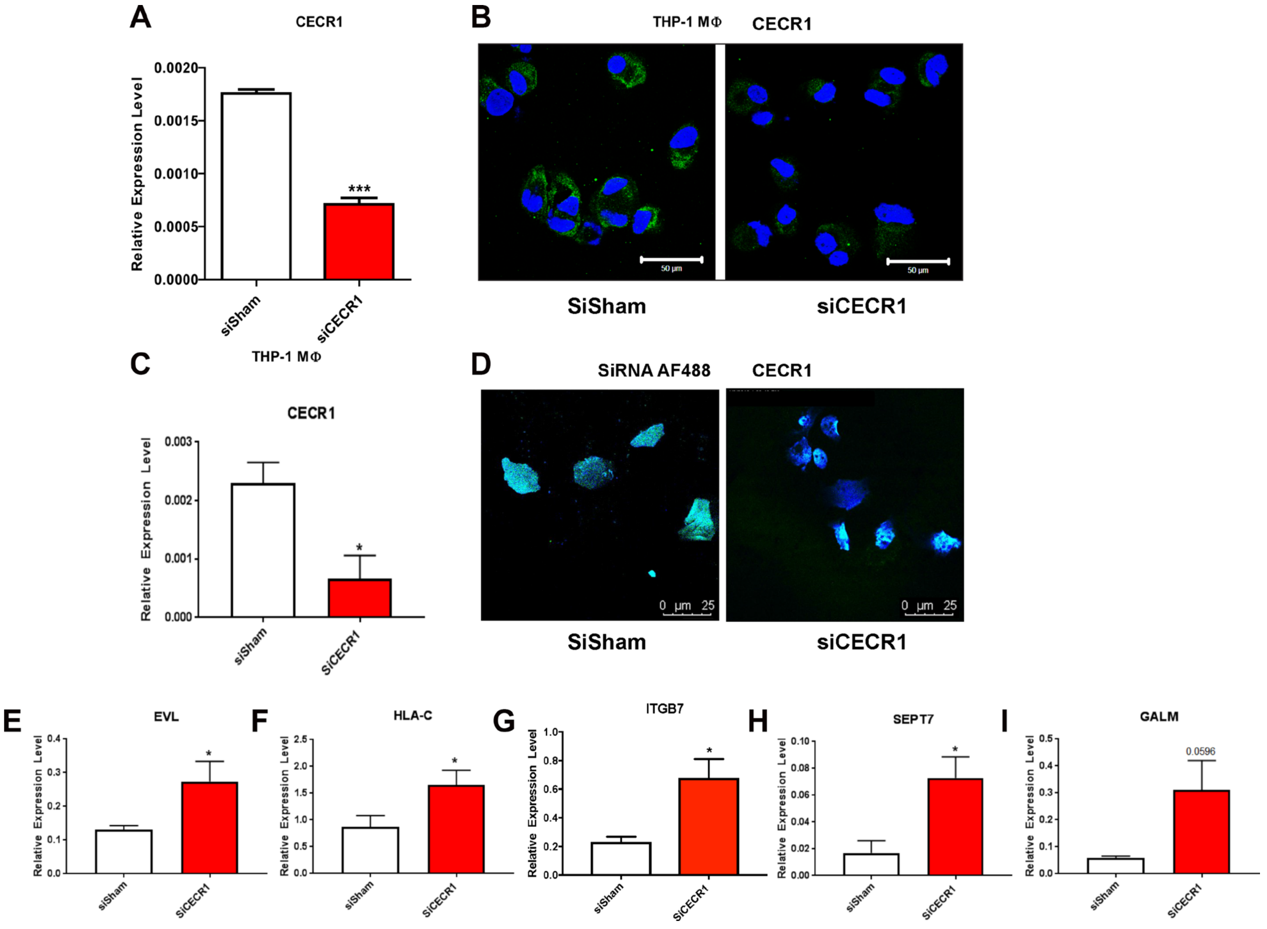


**Supplementary Figure 1:** (**A**) Transcript of CECR1 treated with siRNA (Dharmacon) was assessed by real time qPCR. Data were shown as Mean ± SEM. ***$P < 0.005$ based on student's $T$-test. $N = 3$. (**B**) Representative figure of CECR1 immunostaining of macrophages with siSham and siCECR1 (Dharmacon) transfection. Experiments were repeated three times with similar read out. Scale bar: 50 µm. (**C**) Transcript of CECR1 treated with siRNA (Sigma) was assessed by real time qPCR. Data were shown as Mean ± SEM. *$P < 0.05$ based on student's $T$-test. $N = 5$. (**D**) Representative figure of CECR1 immunostaining of macrophages with siSham and siCECR1(Sigma) transfection. Experiments were repeated three times with similar read out. Scale bar: 25 µm. (**E–I**) Transcripts of EVL (E), HLA-C (F), ITGB7 (G), SEPT7 (H) and GALM (I) after siRNA (Sigma) treatment were assessed by real time qPCR. Data were shown as Mean ± SEM. *$P < 0.05$ based on student's $T$-test. $N = 5$..

## IFN-γ Responsive Genes

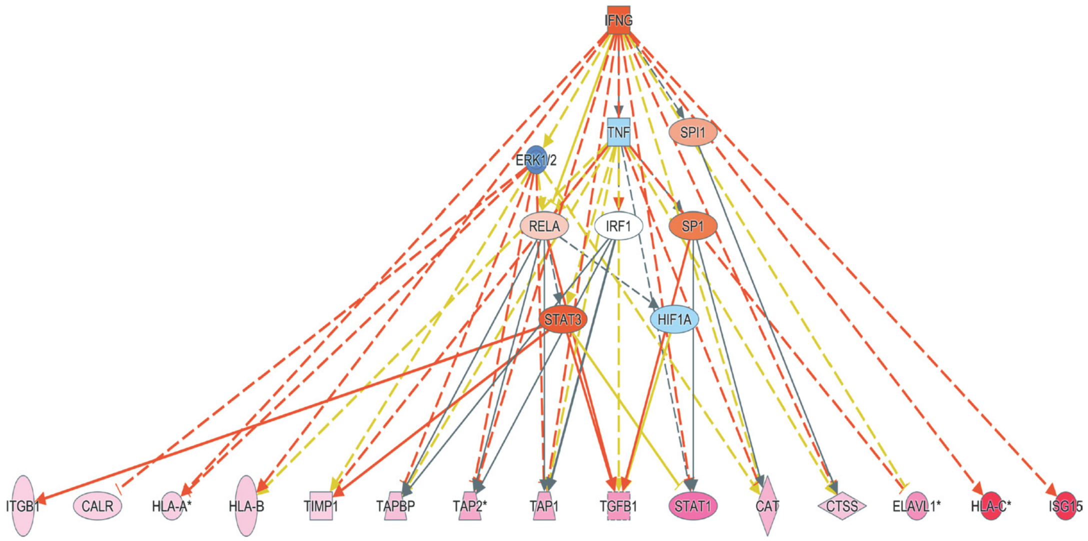


**Supplementary Figure 2: IPA diagram showing proteins enriched in siCECR1 macrophages that are predicated to be the downstream molecules of IFN-γ signaling pathway.**

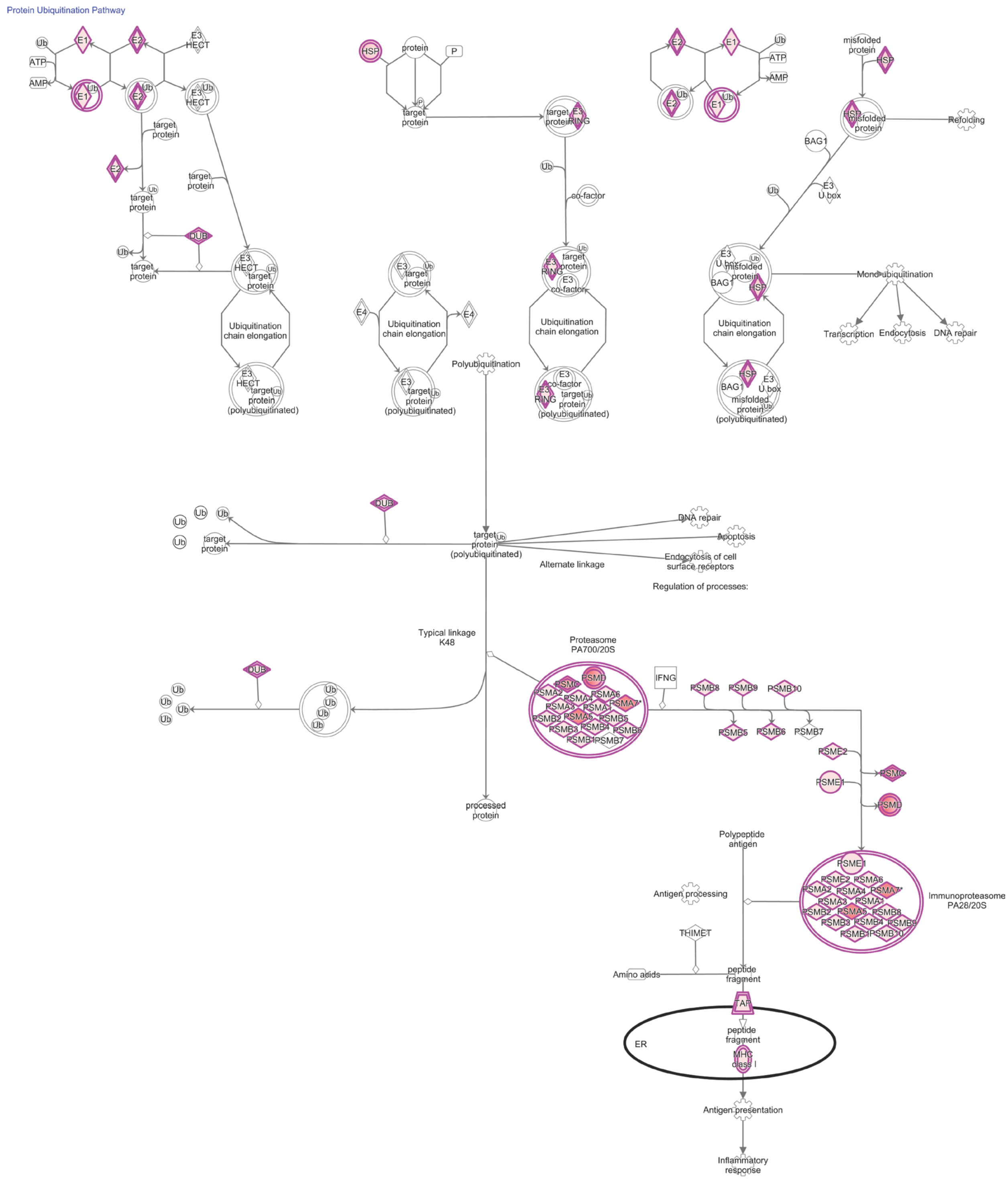


**Supplementary Figure 3: Schematic diagram of IPA data showing involvement of PSMA5, 7, PSMC6, PSMD8 in S20 proteasome complex related pathways.**

**Supplementary Table 1: Significantly upregulated proteins in MΦ siSham compared to MΦ siCECR1**

| UniProt | Symbol | Entrez gene name | Location | Type(s) | Fold change | *P*-value |
|---|---|---|---|---|---|---|
| O43312 | MTSS1 | metastasis suppressor 1 | Cytoplasm | transporter | 4.33 | 0.001 |
| Q8IXB1 | DNAJC10 | DnaJ heat shock protein family (Hsp40) member C10 | Cytoplasm | enzyme | 3.33 | 0.002 |
| Q8NE86 | MCU | mitochondrial calcium uniporter | Cytoplasm | enzyme | 3.33 | 0.002 |
| P62899 | RPL31 | ribosomal protein L31 | Cytoplasm | enzyme | 3.33 | 0.002 |
| P63313 | TMSB10 | thymosin beta 10 | Cytoplasm | phosphatase | 3.33 | 0.001 |
| Q99471 | PFDN5 | prefoldin subunit 5 | Nucleus | enzyme | 3.00 | 0.001 |
| Q9UBQ0 | VPS29 | VPS29 retromer complex component | Cytoplasm | other | 3.00 | 0.001 |
| P06702 | S100A9 | S100 calcium binding protein A9 | Cytoplasm | enzyme | 2.40 | 0.018 |
| P62333 | PSMC6 | proteasome 26S subunit. ATPase 6 | Nucleus | kinase | 1.86 | 0.035 |
| Q9BUJ2 | HNRNPUL1 | heterogeneous nuclear ribonucleoprotein U like 1 | Nucleus | kinase | 1.70 | 0.035 |
| Q14643 | ITPR1 | inositol 1.4.5-trisphosphate receptor type 1 | Cytoplasm | other | 1.60 | 0.016 |
| P09668 | CTSH | cathepsin H | Cytoplasm | other | 1.56 | 0.007 |
| Q92974 | ARHGEF2 | Rho/Rac guanine nucleotide exchange factor 2 | Cytoplasm | enzyme | 1.47 | 0.033 |
| P14868 | DARS | aspartyl-tRNA synthetase | Cytoplasm | other | 1.36 | 0.034 |
| Q15019 | SEPT2 | septin 2 | Cytoplasm | enzyme | 1.33 | 0.007 |
| Q9NSD9 | FARSB | phenylalanyl-tRNA synthetase beta subunit | Cytoplasm | enzyme | 1.30 | 0.047 |
| Q9UHB9 | SRP68 | signal recognition particle 68 | Nucleus | other | 1.30 | 0.047 |
| P27348 | YWHAQ | tyrosine 3-monooxygenase/tryptophan 5-monooxygenase activation protein theta | Cytoplasm | other | 1.28 | 0.039 |
| P34910 | EVI2B | ecotropic viral integration site 2B | Plasma Membrane | enzyme | 1.27 | 0.016 |
| Q92598 | HSPH1 | heat shock protein family H (Hsp110) member 1 | Cytoplasm | enzyme | 1.26 | 0.004 |
| P09661 | SNRPA1 | small nuclear ribonucleoprotein polypeptide A’ | Nucleus | other | 1.25 | 0.007 |
| P52815 | MRPL12 | mitochondrial ribosomal protein L12 | Cytoplasm | enzyme | 1.24 | 0.016 |
| O43399 | TPD52L2 | tumor protein D52 like 2 | Cytoplasm | other | 1.23 | 0.025 |
| Q14697 | GANAB | glucosidase II alpha subunit | Cytoplasm | transcription regulator | 1.14 | 0.04 |
| P08559 | PDHA1 | pyruvate dehydrogenase (lipoamide) alpha 1 | Cytoplasm | phosphatase | 1.11 | 0.026 |
| P49368 | CCT3 | chaperonin containing TCP1 subunit 3 | Cytoplasm | transcription regulator | 1.11 | 0.033 |
| P07339 | CTSD | cathepsin D | Cytoplasm | peptidase | 1.11 | 0.05 |
| Q06830 | PRDX1 | peroxiredoxin 1 | Cytoplasm | other | 1.10 | 0.029 |
| Q8TCT9 | HM13 | histocompatibility (minor) 13 | Cytoplasm | other | 1.06 | 0.047 |
| P26641 | EEF1G | eukaryotic translation elongation factor 1 gamma | Cytoplasm | enzyme | 1.05 | 0.015 |
| Q07020 | RPL18 | ribosomal protein L18 | Cytoplasm | enzyme | 1.05 | 0.047 |
| P07355 | ANXA2 | annexin A2 | Plasma Membrane | transporter | 1.05 | 0.028 |
| P14618 | PKM | pyruvate kinase. muscle | Cytoplasm | transporter | 1.01 | 0.003 |

**Supplementary Table 2: Enriched proteins in MΦ siCECR1 compared to MΦ siSham.** See Supplementary_Table_2

**Supplementary Table 3: Significantly upregulated proteins in U87-MΦ siCECR1 compared to U87-MΦ siSham**

| Uniprot | Symbol | Entrez gene name | Location | Type(s) | Fold change | *P*-Value |
|---|---|---|---|---|---|---|
| P49757 | NUMB | NUMB endocytic adaptor protein | Plasma Membrane | other | 3.665474579 | 0.001 |
| Q13231 | CHIT1 | chitinase 1 | Extracellular Space | enzyme | 3.333412829 | 0.001 |
| P01137 | TGFB1 | transforming growth factor beta 1 | Extracellular Space | growth factor | 3.000077979 | 0.019 |
| Q16181 | SEPT7 | septin 7 | Cytoplasm | other | 2.500124605 | 0.038 |
| Q9GZY6 | LAT2 | linker for activation of T-cells family member 2 | Plasma Membrane | other | 2 | 0.047 |
| P40429 | RPL13A | ribosomal protein L13a | Cytoplasm | other | 1.777685362 | 0.039 |
| P30041 | PRDX6 | peroxiredoxin 6 | Cytoplasm | enzyme | 1.624504793 | 0.033 |
| P50281 | MMP14 | matrix metallopeptidase 14 | Extracellular Space | peptidase | 1.500038989 | 0.007 |
| P30520 | ADSS | adenylosuccinate synthase | Cytoplasm | enzyme | 1.461044379 | 0.047 |
| P49327 | FASN | fatty acid synthase | Cytoplasm | enzyme | 1.382232207 | 0.006 |
| Q9BV40 | VAMP8 | vesicle associated membrane protein 8 | Plasma Membrane | transporter | 1.363202607 | 0.013 |
| P08559 | PDHA1 | pyruvate dehydrogenase (lipoamide) alpha 1 | Cytoplasm | enzyme | 1.278099363 | 0.047 |
| Q07955 | SRSF1 | serine/arginine-rich splicing factor 1 | Nucleus | other | 1.266634254 | 0.016 |
| P26038 | MSN | moesin | Plasma Membrane | other | 1.20664392 | 0.038 |
| O00567 | NOP56 | NOP56 ribonucleoprotein | Nucleus | other | 1.17609125 | 0.026 |
| P18669 | PGAM1 | phosphoglycerate mutase 1 | Cytoplasm | phosphatase | 1.135242102 | 0.016 |
| P07900 | HSP90AA1 | heat shock protein 90kDa alpha family class A member 1 | Cytoplasm | enzyme | 1.100378609 | 0.023 |
| P62937 | PPIA | peptidylprolyl isomerase A | Cytoplasm | enzyme | 1.060687741 | 0.001 |

**Supplementary Table 4: Significantly downregulated proteins in U87-MΦ siCECR1 compared to U87-MΦ siSham**

| Uniprot | Symbol | Entrez gene name | Location | Type(s) | Fold change | *P*-Value |
|---|---|---|---|---|---|---|
| Q0VD83 | APOBR | apolipoprotein B receptor | Plasma Membrane | transmembrane receptor | 0.21 | 0.002 |
| O94874 | UFL1 | UFM1 specific ligase 1 | Cytoplasm | other | 0.25 | 0.006 |
| Q8N1F7 | NUP93 | nucleoporin 93kDa | Nucleus | other | 0.30 | 0.001 |
| Q99685 | MGLL | monoglyceride lipase | Plasma Membrane | enzyme | 0.30 | 0.001 |
| P48556 | PSMD8 | proteasome 26S subunit. non-ATPase 8 | Cytoplasm | other | 0.30 | 0.006 |
| P00749 | PLAU | plasminogen activator. urokinase | Extracellular Space | peptidase | 0.30 | 0.04 |
| P54886 | ALDH18A1 | aldehyde dehydrogenase 18 family member A1 | Cytoplasm | kinase | 0.33 | 0.002 |
| Q14011 | CIRBP | cold inducible RNA binding protein | Nucleus | translation regulator | 0.33 | 0.002 |
| P53634 | CTSC | cathepsin C | Cytoplasm | peptidase | 0.33 | 0.002 |
| Q8IXB1 | DNAJC10 | DnaJ heat shock protein family (Hsp40) member C10 | Cytoplasm | enzyme | 0.40 | 0.034 |
| P62979 | RPS27A | ribosomal protein S27a | Cytoplasm | other | 0.47 | 0.049 |
| P28066 | PSMA5 | proteasome subunit alpha 5 | Cytoplasm | peptidase | 0.59 | 0.007 |
| Q96JJ7 | TMX3 | thioredoxin related transmembrane protein 3 | Cytoplasm | enzyme | 0.64 | 0.001 |
| Q5JRX3 | PITRM1 | pitrilysin metallopeptidase 1 | Cytoplasm | peptidase | 0.65 | 0.025 |
| O94925 | GLS | glutaminase | Cytoplasm | enzyme | 0.70 | 0.016 |
| O96008 | TOMM40 | translocase of outer mitochondrial membrane 40 | Cytoplasm | ion channel | 0.72 | 0.034 |
| P23284 | PPIB | peptidylprolyl isomerase B | Cytoplasm | enzyme | 0.73 | 0.02 |
| Q00341 | HDLBP | high density lipoprotein binding protein | Nucleus | transporter | 0.74 | 0.024 |
| P07305 | H1F0 | H1 histone family member 0 | Nucleus | other | 0.74 | 0.025 |
| P00491 | PNP | purine nucleoside phosphorylase | Nucleus | enzyme | 0.74 | 0.047 |
| P62333 | PSMC6 | proteasome 26S subunit. ATPase 6 | Nucleus | peptidase | 0.75 | 0.007 |
| O14818 | PSMA7 | proteasome subunit alpha 7 | Cytoplasm | peptidase | 0.75 | 0.007 |
| P43490 | NAMPT | nicotinamide phosphoribosyltransferase | Extracellular Space | cytokine | 0.75 | 0.031 |
| P10515 | DLAT | dihydrolipoamide S-acetyltransferase | Cytoplasm | enzyme | 0.79 | 0.047 |
| P99999 | CYCS | cytochrome c. somatic | Cytoplasm | transporter | 0.80 | 0.024 |
| O95571 | ETHE1 | ETHE1. persulfide dioxygenase | Cytoplasm | enzyme | 0.87 | 0.033 |
| Q15942 | ZYX | zyxin | Plasma Membrane | other | 0.88 | 0.047 |
| P61019 | RAB2A | RAB2A. member RAS oncogene family | Cytoplasm | enzyme | 0.92 | 0.026 |
| P62277 | RPS13 | ribosomal protein S13 | Cytoplasm | other | 0.95 | 0.025 |

**Supplementary Table 5: Upregulated proteins in U87-MΦ siSham compared to MΦ siSham.** See Supplementary_Table_5

**Supplementary Table 6: Downregulated proteins in U87-MΦ siSham compared to MΦ siSham.** See Supplementary_Table_6

**Supplementary Table 7: Sequences of Primers used for Real time qPCR**

| Primers | | Oligo sequence (5′-3′) |
|---|---|---|
| **β-actin** | Forward | TCCCTGGAGAAGAGCTACGA |
| **β-actin** | Reverse | AGCACTGTGTTGGCGTACAG |
| **CECR1** | Forward | TGGCGTTAAGCTGCCTTACT |
| **CECR1** | Reverse | GCTACAGGGTGGTTCCTCAA |
| **CTSH** | Forward | CCTGTGAAAAATCAGGGTGCCT |
| **CTSH** | Reverse | TGAAGACAACTGAGGCTGCAA |
| **EVL** | Forward | AGGGTCTGTGGTCCTCTGAT |
| **EVL** | Reverse | GCAGATACTCTGTTCACTTGTGG |
| **GALM** | Forward | CCAGTTCTCGCGCATCAGT |
| **GALM** | Reverse | GCCATCCAGGGTGTATGTCA |
| **HLA-A** | Forward | CGACGCCGCGAGCCAGA |
| **HLA-A** | Reverse | GCGATGTAATCCTTGCCGTCGTAG |
| **HLA-C** | Forward | GGAGACACAGAAGTACAAGCG |
| **HLA-C** | Reverse | CGTCGTAGGCGTACTGGTCATA |
| **IL-10** | Forward | GGCACCCAGTCTGAGAACAG |
| **IL-10** | Reverse | TGGCAACCCAGGTAACCCTTA |
| **IL-12p35** | Forward | GCTCCAGAAGGCCAGACAAA |
| **IL-12p35** | Reverse | GCCAGGCAACTCCCATTAGT |
| **ISG15** | Forward | CGCAGATCACCCAGAAGATCG |
| **ISG15** | Reverse | TTCGTCGCATTTGTCCACCA |
| **ITGB7** | Forward | GAGGAAGGACTGCTCTGCAC |
| **ITGB7** | Reverse | ACCATGCCCGAGATCCCAAG |
| **PLAU** | Forward | CCCAGGAAATGGGACAGGG |
| **PLAU** | Reverse | ACAGTTCGCCTGTTCGTATCT |
| **Sept7** | Forward | CACGCTTATGGTAGTGGGTGA |
| **Sept7** | Reverse | TGAGCAGCAACTGAACACCA |
| **S100A9** | Forward | TCCTCGGCTTTGACAGAGTG |
| **S100A9** | Reverse | TGCCCCAGCTTCACAGAGTA |
| **WDFY** | Forward | ACGGATATTTGTGGGCCAGG |
| **WDFY** | Reverse | CGGTTCTGATGAGCTGGGTA |
