# Supplementary material for: Comparative proteomic analysis of cat eye syndrome critical region protein 1- function in tumor-associated macrophages and immune response regulation of glial tumors
Source: Oncotarget. 2018 Sep 11;9(71):33500–14. doi: 10.18632/oncotarget.26063 (PMC6173361; doi:10.18632/oncotarget.26063)
Supplement: Supplementary file 2 [file oncotarget-09-33500-s002.docx]

**Supplementary Table 2: Enriched proteins in MФ siCECR1 compared to MФ siSham**

| **Uniprot** | **Symbol** | **Entrez Gene Name** | **Location** | **Type(s)** | **Fold Change** | **P-value** |
| --- | --- | --- | --- | --- | --- | --- |
| P30501 | HLA-C | major histocompatibility complex. class I. C | Plasma Membrane | enzyme | 8.33 | 0.001 |
| Q16181 | SEPT7 | septin 7 | Cytoplasm | transporter | 5.33 | 0.007 |
| P26010 | ITGB7 | integrin subunit beta 7 | Plasma Membrane | transporter | 4.33 | 0.001 |
| P05161 | ISG15 | ISG15 ubiquitin-like modifier | Extracellular Space | enzyme | 4.00 | 0.008 |
| Q9UI08 | EVL | Enah/Vasp-like | Plasma Membrane | enzyme | 3.67 | 0.001 |
| Q96C23 | GALM | galactose mutarotase (aldose 1-epimerase) | Cytoplasm | enzyme | 3.67 | 0.001 |
| Q53H82 | LACTB2 | lactamase beta 2 | Cytoplasm | enzyme | 3.67 | 0.006 |
| Q8IWB7 | WDFY1 | WD repeat and FYVE domain containing 1 | Cytoplasm | other | 3.67 | 0.001 |
| P00390 | GSR | glutathione reductase | Cytoplasm | transcription regulator | 3.00 | 0.029 |
| Q96N66 | MBOAT7 | membrane bound O-acyltransferase domain containing 7 | Plasma Membrane | other | 3.00 | 0.001 |
| Q9NX40 | OCIAD1 | OCIA domain containing 1 | Cytoplasm | other | 3.00 | 0.003 |
| Q13596 | SNX1 | sorting nexin 1 | Cytoplasm | enzyme | 3.00 | 0.026 |
| Q0VD83 | APOBR | apolipoprotein B receptor | Plasma Membrane | enzyme | 2.83 | 0.026 |
| Q8TCS8 | PNPT1 | polyribonucleotide nucleotidyltransferase 1 | Cytoplasm | other | 2.75 | 0.029 |
| Q92804 | TAF15 | TATA-box binding protein associated factor 15 | Nucleus | transcription regulator | 2.00 | 0.047 |
| P54920 | NAPA | NSF attachment protein alpha | Cytoplasm | peptidase | 1.83 | 0.005 |
| Q15717 | ELAVL1 | ELAV like RNA binding protein 1 | Cytoplasm | other | 1.78 | 0.025 |
| P43304 | GPD2 | glycerol-3-phosphate dehydrogenase 2 | Cytoplasm | other | 1.75 | 0.042 |
| P01137 | TGFB1 | transforming growth factor beta 1 | Extracellular Space | translation regulator | 1.67 | 0.008 |
| Q9NYU2 | UGGT1 | UDP-glucose glycoprotein glucosyltransferase 1 | Cytoplasm | translation regulator | 1.67 | 0.011 |
| Q8NBQ5 | HSD17B11 | hydroxysteroid (17-beta) dehydrogenase 11 | Cytoplasm | other | 1.64 | 0.025 |
| P29218 | IMPA1 | inositol monophosphatase 1 | Cytoplasm | translation regulator | 1.60 | 0.035 |
| Q02543 | RPL18A | ribosomal protein L18a | Cytoplasm | enzyme | 1.58 | 0.025 |
| Q00688 | FKBP3 | FK506 binding protein 3 | Nucleus | other | 1.56 | 0.016 |
| Q02978 | SLC25A11 | solute carrier family 25 member 11 | Cytoplasm | enzyme | 1.56 | 0.047 |
| O95466 | FMNL1 | formin like 1 | Cytoplasm | cytokine | 1.50 | 0.025 |
| Q9UGP8 | SEC63 | SEC63 homolog. protein translocation regulator | Cytoplasm | enzyme | 1.50 | 0.023 |
| Q03518 | TAP1 | transporter 1. ATP-binding cassette. sub-family B (MDR/TAP) | Cytoplasm | other | 1.50 | 0.006 |
| P11413 | G6PD | glucose-6-phosphate dehydrogenase | Cytoplasm | transmembrane receptor | 1.48 | 0.013 |
| P16435 | POR | cytochrome p450 oxidoreductase | Cytoplasm | transmembrane receptor | 1.48 | 0.01 |
| Q9P2E9 | RRBP1 | ribosome binding protein 1 | Cytoplasm | enzyme | 1.46 | 0.016 |
| P14854 | COX6B1 | cytochrome c oxidase subunit 6B1 | Cytoplasm | enzyme | 1.45 | 0.013 |
| Q03519 | TAP2 | transporter 2. ATP-binding cassette. sub-family B (MDR/TAP) | Cytoplasm | other | 1.44 | 0.005 |
| P04040 | CAT | catalase | Cytoplasm | enzyme | 1.43 | 0.002 |
| P62280 | RPS11 | ribosomal protein S11 | Cytoplasm | other | 1.42 | 0.016 |
| P62820 | RAB1A | RAB1A. member RAS oncogene family | Cytoplasm | enzyme | 1.41 | 0.038 |
| O14950 | MYL12B | myosin light chain 12B | Cytoplasm | enzyme | 1.38 | 0.001 |
| Q9Y3Z3 | SAMHD1 | SAM domain and HD domain 1 | Nucleus | enzyme | 1.36 | 0.015 |
| P27105 | STOM | stomatin | Plasma Membrane | enzyme | 1.36 | 0.025 |
| P07305 | H1F0 | H1 histone family member 0 | Nucleus | enzyme | 1.33 | 0.013 |
| O15533 | TAPBP | TAP binding protein (tapasin) | Cytoplasm | kinase | 1.33 | 0.023 |
| Q13488 | TCIRG1 | T-cell immune regulator 1. ATPase H+ transporting V0 subunit a3 | Plasma Membrane | kinase | 1.33 | 0.007 |
| O75947 | ATP5H | ATP synthase. H+ transporting. mitochondrial Fo complex subunit D | Cytoplasm | peptidase | 1.31 | 0.026 |
| P25774 | CTSS | cathepsin S | Cytoplasm | enzyme | 1.30 | 0.025 |
| P07737 | PFN1 | profilin 1 | Cytoplasm | other | 1.30 | 0.018 |
| P31948 | STIP1 | stress induced phosphoprotein 1 | Cytoplasm | ion channel | 1.28 | 0.013 |
| P23786 | CPT2 | carnitine palmitoyltransferase 2 | Cytoplasm | enzyme | 1.28 | 0.021 |
| P10319 | HLA-B | major histocompatibility complex. class I. B | Plasma Membrane | transmembrane receptor | 1.28 | 0.035 |
| P13804 | ETFA | electron transfer flavoprotein alpha subunit | Cytoplasm | enzyme | 1.27 | 0.018 |
| O75874 | IDH1 | isocitrate dehydrogenase (NADP(+)) 1. cytosolic | Cytoplasm | transcription regulator | 1.26 | 0.005 |
| P22307 | SCP2 | sterol carrier protein 2 | Cytoplasm | transmembrane receptor | 1.25 | 0.033 |
| P01892 | HLA-A | major histocompatibility complex. class I. A | Plasma Membrane | ion channel | 1.24 | 0.007 |
| Q99536 | VAT1 | vesicle amine transport 1 | Plasma Membrane | transporter | 1.23 | 0.024 |
| P05556 | ITGB1 | integrin subunit beta 1 | Plasma Membrane | other | 1.19 | 0.05 |
| Q9NX63 | CHCHD3 | coiled-coil-helix-coiled-coil-helix domain containing 3 | Cytoplasm | enzyme | 1.19 | 0.047 |
| P46459 | NSF | N-ethylmaleimide sensitive factor | Cytoplasm | enzyme | 1.17 | 0.031 |
| Q99623 | PHB2 | prohibitin 2 | Cytoplasm | other | 1.15 | 0.039 |
| P27797 | CALR | calreticulin | Cytoplasm | other | 1.14 | 0.01 |
| P55265 | ADAR | adenosine deaminase. RNA-specific | Nucleus | peptidase | 1.13 | 0.013 |
| Q15942 | ZYX | zyxin | Plasma Membrane | enzyme | 1.12 | 0.006 |
| P12109 | COL6A1 | collagen type VI alpha 1 | Extracellular Space | peptidase | 1.10 | 0.047 |
| Q02878 | RPL6 | ribosomal protein L6 | Nucleus | other | 1.10 | 0.024 |
| P10599 | TXN | thioredoxin | Cytoplasm | other | 1.07 | 0.007 |
| P07910 | HNRNPC | heterogeneous nuclear ribonucleoprotein C (C1/C2) | Nucleus | other | 1.06 | 0.007 |
| P28799 | GRN | granulin | Extracellular Space | other | 1.06 | 0.016 |
| Q15067 | ACOX1 | acyl-CoA oxidase 1. palmitoyl | Cytoplasm | other | 1.05 | 0.047 |
| P01033 | TIMP1 | TIMP metallopeptidase inhibitor 1 | Extracellular Space | other | 1.05 | 0.013 |
