# Supplementary material for: Comparative proteomic analysis of cat eye syndrome critical region protein 1- function in tumor-associated macrophages and immune response regulation of glial tumors
Source: Oncotarget. 2018 Sep 11;9(71):33500–14. doi: 10.18632/oncotarget.26063 (PMC6173361; doi:10.18632/oncotarget.26063)
Supplement: Supplementary file 3 [file oncotarget-09-33500-s003.docx]

| **Supplementary Table 5: Upregulated proteins in U87-MФ siSham compared to MФ siSham** | | | | | | |
| --- | --- | --- | --- | --- | --- | --- |
| **Uniprot** | **Symbol** | **Entrez Gene Name** | **Location** | **Type(s)** | **Fold Change** | **P-Value** |
| P01584 | IL1B | interleukin 1 beta | Extracellular Space | cytokine | 7.00 | 0.002 |
| O75431 | MTX2 | metaxin 2 | Cytoplasm | transporter | 4.33 | 0.008 |
| Q9Y2J2 | EPB41L3 | erythrocyte membrane protein band 4.1 like 3 | Plasma Membrane | other | 3.50 | 0.009 |
| Q9ULC5 | ACSL5 | acyl-CoA synthetase long-chain family member 5 | Cytoplasm | enzyme | 3.33 | 0.001 |
| P17813 | ENG | endoglin | Plasma Membrane | transmembrane receptor | 3.33 | 0.002 |
| P00749 | PLAU | plasminogen activator. urokinase | Extracellular Space | peptidase | 3.33 | 0.04 |
| P00390 | GSR | glutathione reductase | Cytoplasm | enzyme | 3.20 | 0.008 |
| P28074 | PSMB5 | proteasome subunit beta 5 | Cytoplasm | peptidase | 3.00 | 0.002 |
| P54886 | ALDH18A1 | aldehyde dehydrogenase 18 family member A1 | Cytoplasm | kinase | 3.00 | 0.002 |
| P06280 | GLA | galactosidase alpha | Cytoplasm | enzyme | 3.00 | 0.006 |
| P49006 | MARCKSL1 | MARCKS-like 1 | Cytoplasm | other | 3.00 | 0.029 |
| Q9HCC0 | MCCC2 | methylcrotonoyl-CoA carboxylase 2 | Cytoplasm | enzyme | 2.60 | 0.033 |
| Q9UNM6 | PSMD13 | proteasome 26S subunit. non-ATPase 13 | Cytoplasm | peptidase | 2.43 | 0.006 |
| P20340 | RAB6A | RAB6A. member RAS oncogene family | Cytoplasm | enzyme | 2.17 | 0.024 |
| Q9Y230 | RUVBL2 | RuvB like AAA ATPase 2 | Nucleus | transcription regulator | 1.88 | 0.019 |
| Q13148 | TARDBP | TAR DNA binding protein | Nucleus | transcription regulator | 1.88 | 0.047 |
| P35914 | HMGCL | 3-hydroxymethyl-3-methylglutaryl-CoA lyase | Cytoplasm | enzyme | 1.86 | 0.039 |
| P05109 | S100A8 | S100 calcium binding protein A8 | Cytoplasm | other | 1.78 | 0.006 |
| P18031 | PTPN1 | protein tyrosine phosphatase. non-receptor type 1 | Cytoplasm | phosphatase | 1.73 | 0.05 |
| P07305 | H1F0 | H1 histone family member 0 | Nucleus | other | 1.58 | 0.016 |
| Q02543 | RPL18A | ribosomal protein L18a | Cytoplasm | other | 1.58 | 0.016 |
| P06702 | S100A9 | S100 calcium binding protein A9 | Cytoplasm | other | 1.58 | 0.039 |
| P62269 | RPS18 | ribosomal protein S18 | Cytoplasm | other | 1.56 | 0.05 |
| O75533 | SF3B1 | splicing factor 3b subunit 1 | Nucleus | other | 1.54 | 0.009 |
| Q8IXB1 | DNAJC10 | DnaJ heat shock protein family (Hsp40) member C10 | Cytoplasm | enzyme | 1.50 | 0.013 |
| Q5JRX3 | PITRM1 | pitrilysin metallopeptidase 1 | Cytoplasm | peptidase | 1.48 | 0.039 |
| P10515 | DLAT | dihydrolipoamide S-acetyltransferase | Cytoplasm | enzyme | 1.46 | 0.013 |
| P14854 | COX6B1 | cytochrome c oxidase subunit 6B1 | Cytoplasm | enzyme | 1.45 | 0.013 |
| Q9P2E9 | RRBP1 | ribosome binding protein 1 | Cytoplasm | other | 1.43 | 0.009 |
| P62820 | RAB1A | RAB1A. member RAS oncogene family | Cytoplasm | enzyme | 1.41 | 0.001 |
| P49748 | ACADVL | acyl-CoA dehydrogenase. very long chain | Cytoplasm | enzyme | 1.41 | 0.034 |
| P48163 | ME1 | malic enzyme 1 | Cytoplasm | enzyme | 1.40 | 0.007 |
| Q7Z7H5 | TMED4 | transmembrane p24 trafficking protein 4 | Cytoplasm | transporter | 1.40 | 0.047 |
| P51148 | RAB5C | RAB5C. member RAS oncogene family | Cytoplasm | enzyme | 1.38 | 0.019 |
| P23786 | CPT2 | carnitine palmitoyltransferase 2 | Cytoplasm | enzyme | 1.34 | 0.003 |
| Q15067 | ACOX1 | acyl-CoA oxidase 1. palmitoyl | Cytoplasm | enzyme | 1.33 | 0.013 |
| P54920 | NAPA | NSF attachment protein alpha | Cytoplasm | transporter | 1.33 | 0.035 |
| P28066 | PSMA5 | proteasome subunit alpha 5 | Cytoplasm | peptidase | 1.31 | 0.024 |
| P43490 | NAMPT | nicotinamide phosphoribosyltransferase | Extracellular Space | cytokine | 1.29 | 0.003 |
| P13804 | ETFA | electron transfer flavoprotein alpha subunit | Cytoplasm | transporter | 1.24 | 0.003 |
| P11387 | TOP1 | topoisomerase (DNA) I | Nucleus | enzyme | 1.24 | 0.017 |
| P52272 | HNRNPM | heterogeneous nuclear ribonucleoprotein M | Nucleus | other | 1.20 | 0.034 |
| P07602 | PSAP | prosaposin | Extracellular Space | other | 1.13 | 0.006 |
| P25774 | CTSS | cathepsin S | Cytoplasm | peptidase | 1.13 | 0.007 |
| P0DMV8 | HSPA1A/HSPA1B | heat shock protein family A (Hsp70) member 1A | Cytoplasm | enzyme | 1.13 | 0.047 |
| P07910 | HNRNPC | heterogeneous nuclear ribonucleoprotein C (C1/C2) | Nucleus | other | 1.12 | 0 |
| P99999 | CYCS | cytochrome c. somatic | Cytoplasm | transporter | 1.11 | 0.024 |
| P27797 | CALR | calreticulin | Cytoplasm | transcription regulator | 1.10 | 0.049 |
| Q99536 | VAT1 | vesicle amine transport 1 | Plasma Membrane | transporter | 1.09 | 0.013 |
| Q68CZ2 | TNS3 | tensin 3 | Plasma Membrane | phosphatase | 1.07 | 0.03 |
| Q9Y6N5 | SQRDL | sulfide quinone reductase-like (yeast) | Cytoplasm | enzyme | 1.05 | 0.038 |
| P62937 | PPIA | peptidylprolyl isomerase A | Cytoplasm | enzyme | 1.03 | 0.007 |
| P26641 | EEF1G | eukaryotic translation elongation factor 1 gamma | Cytoplasm | translation regulator | 1.02 | 0.019 |
| P06576 | ATP5B | ATP synthase. H+ transporting. mitochondrial F1 complex. beta polypeptide | Cytoplasm | transporter | 1.01 | 0.005 |
