# Supplementary material for: Comparative proteomic analysis of cat eye syndrome critical region protein 1- function in tumor-associated macrophages and immune response regulation of glial tumors
Source: Oncotarget. 2018 Sep 11;9(71):33500–14. doi: 10.18632/oncotarget.26063 (PMC6173361; doi:10.18632/oncotarget.26063)
Supplement: Supplementary file 4 [file oncotarget-09-33500-s004.docx]

**Supplementary Table 6: Downregulated proteins in U87-MФ siSham compared to MФ siSham**

| **UniProt** | **Symbol** | **Entrez Gene Name** | **Location** | **Type(s)** | **Fold Change** | **P-Value** |
| --- | --- | --- | --- | --- | --- | --- |
| P60709 | ACTB | actin. beta | Cytoplasm | other | 0.99 | 0.039 |
| P06733 | ENO1 | enolase 1 | Cytoplasm | enzyme | 0.99 | 0.032 |
| P49368 | CCT3 | chaperonin containing TCP1 subunit 3 | Cytoplasm | other | 0.97 | 0.024 |
| Q9Y490 | TLN1 | talin 1 | Plasma Membrane | other | 0.97 | 0.026 |
| P04075 | ALDOA | aldolase. fructose-bisphosphate A | Cytoplasm | enzyme | 0.97 | 0.043 |
| P07437 | TUBB | tubulin beta class I | Cytoplasm | other | 0.96 | 0.03 |
| P07355 | ANXA2 | annexin A2 | Plasma Membrane | other | 0.94 | 0.033 |
| Q15365 | PCBP1 | poly(rC) binding protein 1 | Nucleus | translation regulator | 0.94 | 0.046 |
| P40121 | CAPG | capping actin protein. gelsolin like | Nucleus | other | 0.91 | 0.002 |
| Q06830 | PRDX1 | peroxiredoxin 1 | Cytoplasm | enzyme | 0.91 | 0.034 |
| Q06323 | PSME1 | proteasome activator subunit 1 | Cytoplasm | other | 0.90 | 0.013 |
| Q9BQE3 | TUBA1C | tubulin alpha 1c | Cytoplasm | other | 0.90 | 0.003 |
| P18669 | PGAM1 | phosphoglycerate mutase 1 | Cytoplasm | phosphatase | 0.88 | 0.043 |
| P07900 | HSP90AA1 | heat shock protein 90kDa alpha family class A member 1 | Cytoplasm | enzyme | 0.88 | 0.027 |
| P27348 | YWHAQ | tyrosine 3-monooxygenase/tryptophan 5-monooxygenase activation protein theta | Cytoplasm | other | 0.86 | 0.003 |
| P52907 | CAPZA1 | capping actin protein of muscle Z-line alpha subunit 1 | Cytoplasm | other | 0.86 | 0.013 |
| Q99798 | ACO2 | aconitase 2 | Cytoplasm | enzyme | 0.85 | 0.016 |
| P18621 | RPL17 | ribosomal protein L17 | Cytoplasm | other | 0.84 | 0.024 |
| P20702 | ITGAX | integrin subunit alpha X | Plasma Membrane | transmembrane receptor | 0.83 | 0.015 |
| P09211 | GSTP1 | glutathione S-transferase pi 1 | Cytoplasm | enzyme | 0.82 | 0.008 |
| P54136 | RARS | arginyl-tRNA synthetase | Cytoplasm | enzyme | 0.82 | 0.016 |
| Q32P28 | P3H1 | prolyl 3-hydroxylase 1 | Nucleus | enzyme | 0.81 | 0.047 |
| P68104 | EEF1A1 | eukaryotic translation elongation factor 1 alpha 1 | Cytoplasm | translation regulator | 0.79 | 0.036 |
| Q92598 | HSPH1 | heat shock protein family H (Hsp110) member 1 | Cytoplasm | other | 0.78 | 0.044 |
| O43312 | MTSS1 | metastasis suppressor 1 | Cytoplasm | other | 0.77 | 0.047 |
| Q9UI12 | ATP6V1H | ATPase H+ transporting V1 subunit H | Cytoplasm | transporter | 0.75 | 0.016 |
| Q16831 | UPP1 | uridine phosphorylase 1 | Cytoplasm | enzyme | 0.73 | 0.05 |
| Q9UQ80 | PA2G4 | proliferation-associated 2G4 | Nucleus | transcription regulator | 0.72 | 0.001 |
| P30153 | PPP2R1A | protein phosphatase 2 regulatory subunit A. alpha | Cytoplasm | phosphatase | 0.71 | 0.033 |
| Q96KP4 | CNDP2 | CNDP dipeptidase 2 (metallopeptidase M20 family) | Cytoplasm | peptidase | 0.71 | 0.014 |
| P63313 | TMSB10/TMSB4X | thymosin beta 10 | Cytoplasm | other | 0.70 | 0.035 |
| P15311 | EZR | ezrin | Plasma Membrane | other | 0.69 | 0.021 |
| O14579 | COPE | coatomer protein complex subunit epsilon | Cytoplasm | transporter | 0.69 | 0.025 |
| Q15185 | PTGES3 | prostaglandin E synthase 3 | Cytoplasm | enzyme | 0.65 | 0.023 |
| P40429 | RPL13A | ribosomal protein L13a | Cytoplasm | other | 0.64 | 0.026 |
| P31146 | CORO1A | coronin 1A | Cytoplasm | other | 0.64 | 0.007 |
| Q8NCW5 | NAXE | NAD(P)HX epimerase | Extracellular Space | enzyme | 0.63 | 0.008 |
| Q15019 | SEPT2 | septin 2 | Cytoplasm | enzyme | 0.63 | 0.039 |
| P30041 | PRDX6 | peroxiredoxin 6 | Cytoplasm | enzyme | 0.62 | 0.034 |
| P49327 | FASN | fatty acid synthase | Cytoplasm | enzyme | 0.60 | 0.009 |
| O60749 | SNX2 | sorting nexin 2 | Cytoplasm | transporter | 0.54 | 0.012 |
| P12955 | PEPD | peptidase D | Cytoplasm | peptidase | 0.54 | 0.033 |
| Q14318 | FKBP8 | FK506 binding protein 8 | Cytoplasm | other | 0.50 | 0.047 |
| Q9GZY6 | LAT2 | linker for activation of T-cells family member 2 | Plasma Membrane | other | 0.47 | 0.034 |
| P14174 | MIF | macrophage migration inhibitory factor (glycosylation-inhibiting factor) | Extracellular Space | cytokine | 0.45 | 0.023 |
| P17858 | PFKL | phosphofructokinase. liver type | Cytoplasm | kinase | 0.34 | 0.02 |
| Q9UL25 | RAB21 | RAB21. member RAS oncogene family | Cytoplasm | enzyme | 0.33 | 0.001 |
| Q14012 | CAMK1 | calcium/calmodulin dependent protein kinase I | Cytoplasm | kinase | 0.33 | 0.002 |
| Q9HB71 | CACYBP | calcyclin binding protein | Nucleus | other | 0.30 | 0.002 |
| Q06210 | GFPT1 | glutamine--fructose-6-phosphate transaminase 1 | Cytoplasm | enzyme | 0.30 | 0.002 |
| P62899 | RPL31 | ribosomal protein L31 | Cytoplasm | other | 0.30 | 0.002 |
| P13473 | LAMP2 | lysosomal associated membrane protein 2 | Plasma Membrane | enzyme | 0.30 | 0.002 |
| Q14257 | RCN2 | reticulocalbin 2 | Cytoplasm | other | 0.30 | 0.002 |
| O95352 | ATG7 | autophagy related 7 | Cytoplasm | enzyme | 0.30 | 0.002 |
| P21291 | CSRP1 | cysteine and glycine rich protein 1 | Nucleus | other | 0.27 | 0.001 |
| P16949 | STMN1 | stathmin 1 | Cytoplasm | other | 0.23 | 0.001 |
| Q13177 | PAK2 | p21 protein (Cdc42/Rac)-activated kinase 2 | Cytoplasm | kinase | 0.23 | 0.007 |
| P13798 | APEH | acylaminoacyl-peptide hydrolase | Cytoplasm | peptidase | 0.23 | 0.014 |
| P98171 | ARHGAP4 | Rho GTPase activating protein 4 | Cytoplasm | other | 0.20 | 0.001 |
